# Medicinal Chemistry: A Key Driver in Achieving the Global Sustainable Development Goals

**DOI:** 10.1021/acs.jmedchem.4c03016

**Published:** 2025-03-20

**Authors:** Bianca Martinengo, Eleonora Diamanti, Elisa Uliassi, Maria Laura Bolognesi

**Affiliations:** Department of Pharmacy and Biotechnology, Alma Mater Studiorum - University of Bologna, Via Belmeloro 6, 40126 Bologna, Italy

## Abstract

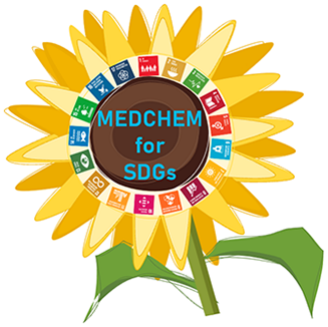

In 2015, the United Nations officially launched the Sustainable Development Goals (SDGs) as “the blueprint to achieve a better and more sustainable future for all. They address the global challenges we face, including those related to poverty, inequality, climate change, environmental degradation, peace and justice. The 17 Goals are all interconnected, in order to leave no one behind, it is important that we achieve them all by 2030”. Here, we have embedded medicinal chemistry as a key player to achieve SDGs. We firmly believe that medicinal chemistry can and must contribute to a sustainable future and a better world with an improved quality of life for all. We have taken a critical look at each of the SDGs, dividing them into Priority and Foundational, and analyzed how medicinal chemistry has an impact on each of them. Although much has been done, we are determined to make progress in this area.

## Significance

- This Perspective highlights the role of medicinal chemistry in advancing key Sustainable Development Goals (SDGs) with a focus on environmental, social, and health impacts.
- It bridges the gap between sustainable practices and the medicinal chemistry community, encouraging the integration of SDGs in current research and innovation.
- It introduces a novel perspective by applying SDG-specific challenges to medicinal chemistry, promoting new approaches to sustainable drug design and production.

## Introduction

We live in a world of an ever-growing population with increasing demands but limited resources. The 2030 Agenda for Sustainable Development, adopted by all United Nations (UN) Member States in 2015,^1^ is a plan of action for people, planet, and prosperity. This ambitious agenda is built upon 17 Sustainable Development Goals (SDGs) that collectively address critical global challenges, ranging from ending poverty and hunger to promoting health, education, and environmental sustainability.^1^ These goals are further broken down into 169 specific targets, providing a clear roadmap to 2030 for tackling the social, economic, and environmental issues facing humanity today.^1^ The SDGs call for a holistic approach to development, recognizing that economic growth must be balanced with social inclusion and environmental protection to achieve sustainable progress. Major challenges such as climate change, inequality, poor health, and unsustainable resource use threaten global well-being, and addressing these requires a concerted, interdisciplinary effort across multiple sectors.

Chemistry is recognized as an integral part of addressing many of the SDGs’ challenges.^2−4^ From improving agricultural practices and water purification techniques to developing renewable energy solutions and creating ecofriendly materials, chemistry provides the foundational knowledge and technologies needed to advance the sustainability agenda. Among the subdisciplines of chemistry, medicinal chemistry, focused on drug discovery and development, stands out for its specific potential to address global health concerns and promote good health and well-being (SDG #3). However, it seems that its reach might extend much further, touching on areas such as poverty reduction, environmental sustainability, and economic growth. As we navigate our way toward a sustainable future, medicinal chemistry might have an essential role to play in this framework, as scientific advances in our field impact, to varying degrees, many of the SDG targets.^5^

Medicinal chemistry is increasingly aligned with sustainability principles.^6−8^ This alignment is driven by the need to address global health challenges, while ensuring environmental protection and promoting sustainable industrial practices. The integration of green and sustainable chemistry principles is crucial in this context, aiming to minimize environmental impact and enhance the efficiency of chemical processes and products.^9,10^

**Figure 1:**
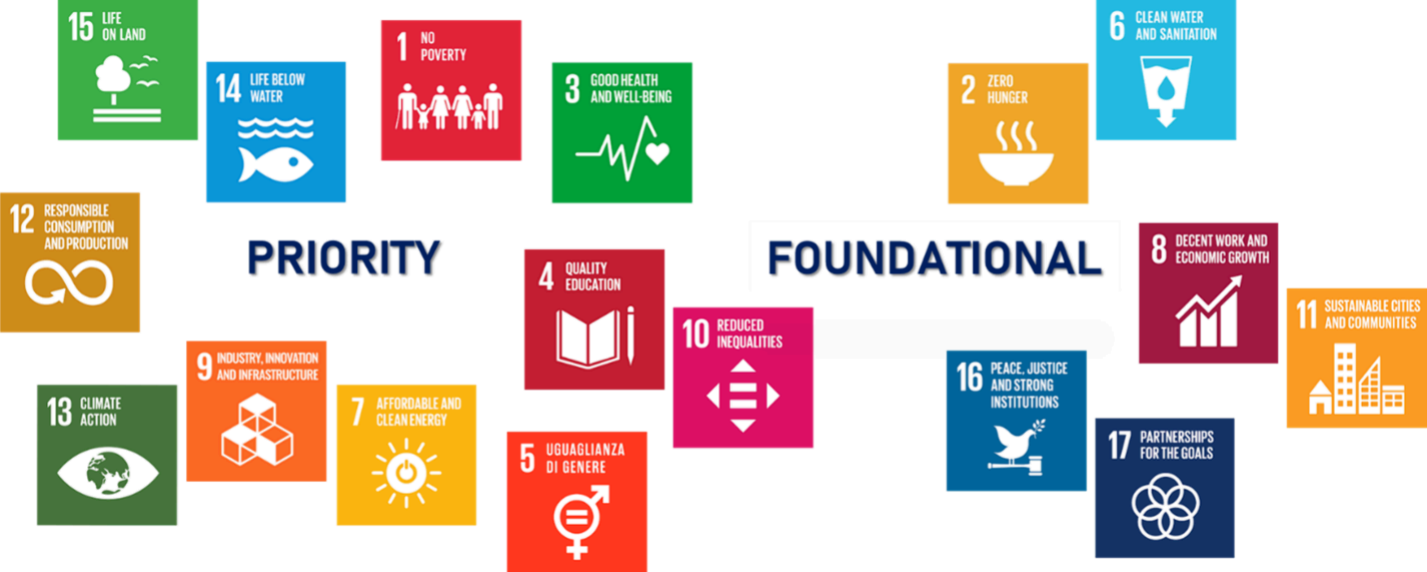
Schematic representation of the 17 SDGs classified into Priority (SDG #1; SDG #3; SDG #4 & SDG #10; SDG #5; SDG #7, SDG #9, & SDG #13; SDG #12; SDG #14 & SDG #15) and Foundational Goals (SDG #2 & SDG #6; SDG #8 & SDG #11; SDG #16 & SDG #17).

In this view and to avoid unnecessary overlap with recent literature dealing with green medicinal chemistry and sustainable drug discovery,^6,7,9−11^ the aim of this Perspective is to critically and punctually analyze the intersection between medicinal chemistry and all the SDGs, emphasizing the significant contributions and potential advancements in this field. Specifically, we will explore relevant case studies where medicinal chemistry has contributed with profound impact to SDGs, categorized as priority goals: No Poverty (SDG #1), Good Health and Well-being (SDG #3), Quality Education (SDG #4) & Reduced Inequalities (SDG #10), Gender Equality (SDG #5), Affordable and Clean Energy (SDG #7), Industry, Innovation, and Infrastructure (SDG #9) & Climate Action (SDG #13), Responsible Consumption and Production (SDG #12), and Life Below Water (SDG #14) & Life on Land (SDG #15) (Figure 1).

Priority SDGs:

- No Poverty (SDG #1): By developing effective, innovative, and affordable medicines targeting Infectious Diseases of Poverty (IDoPs), medicinal chemistry can help break the vicious cycle between illness and disadvantaged situations and combat poverty.
- Good Health and Well-being (SDG #3): This goal specifically focuses on drug discovery and development to combat diseases and enhance human health, thus matching the core values of medicinal chemistry.
- Quality Education (SDG #4) & Reduced Inequalities (SDG #10): Easily accessible learning paths in medicinal chemistry can foster global education and skill development in the field. Ensuring equitable access to scientific education and resources reduces inequalities both within and between countries and is critical for inclusive progress.
- Gender Equality (SDG #5): Recognizing and addressing gender dimensions and inequalities in medicinal chemistry are critical steps in implementing gender mainstreaming as an integral part of sustainable drug discovery activities and beyond.
- Affordable and Clean Energy (SDG #7), Industry, Innovation, and Infrastructure (SDG #9), & Climate Action (SDG #13): Advancements in chemical processes that enhance energy efficiency and develop renewable energy sources are vital. The pharmaceutical industry drives economic growth by creating jobs and fostering innovation. Strengthening research infrastructure and promoting innovative (medicinal) chemistry solutions that address issues affecting climate and the environment contribute to sustainable industrial development.
- Responsible Consumption and Production (SDG #12): Emphasizing green chemistry principles dealing with the use of renewable feedstock, medicinal chemistry can reduce waste and promote sustainable manufacturing processes.
- Life Below Water (SDG #14) & Life on Land (SDG #15): Efforts to minimize pharmaceutical pollutants in aquatic environments protect marine biodiversity. Sustainable land management practices and the development of eco-friendly chemicals support biodiversity and ecosystem health.

In addition to these priority goals, we envisage that the other SDGs can be foundational values for medicinal chemists around the world in their daily practices. They include Zero Hunger (SDG #2); Clean Water and Sanitation (SDG #6); Decent Work and Economic Growth (SDG #8); Sustainable Cities and Communities (SDG #11); Peace, Justice, and Strong Institutions (SDG #16); and Partnership for the Goals (SDG #17) (Figure 1).

In this Perspective, we will delve deeply into each of the priority SDGs and provide a detailed analysis of how medicinal chemistry is poised to contribute to sustainable development through selected case studies. Then, we briefly discuss the foundational ones. Through this exploration, our aim is to offer critical insights and strategic directions for future progress in the field.

**SDG #1**. **No Poverty**. *“End poverty in all its forms everywhere*.*”*^1^

**Figure 2:**
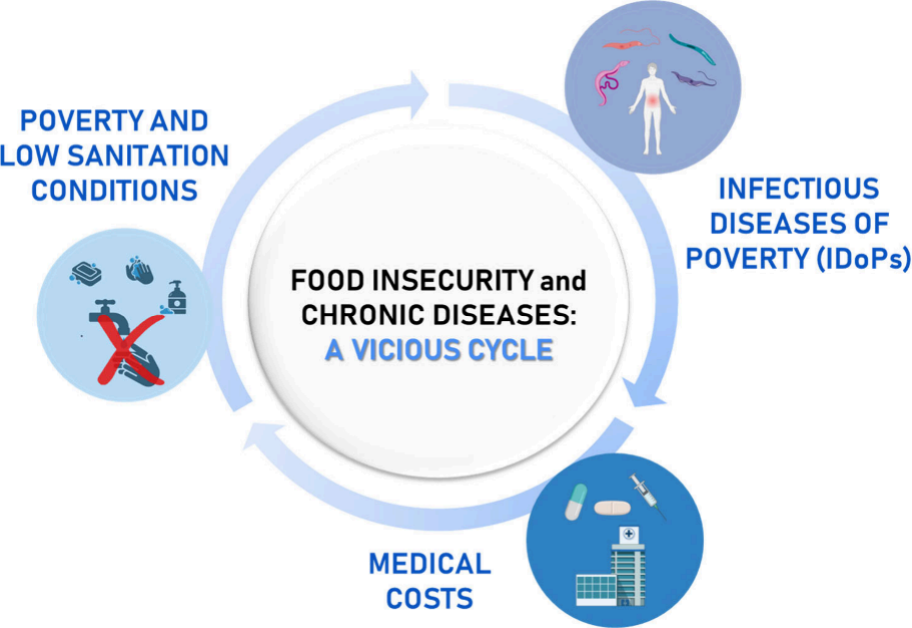
Vicious cycle of poverty-related infectious diseases. This figure was created with BioRender.com.

The eradication of extreme poverty is a pivotal goal of the 2030 Agenda. Back in 1990, a positive trend was seen with more than one billion people that escaped poverty; however, the COVID-19 pandemic reversed it, forcing a million people below the extreme-poverty line.^12^ IDoPs, including Neglected Tropical Diseases (NTDs), continue to impose disproportionate human, social, and economic burden. This is driven by the widespread presence of infectious agents with transmission heavily influenced by socioeconomic and environmental factors. Besides the inadequacy of sanitation conditions and food insecurity, the lack of safe, effective, and affordable medicines is also identified as a key factor that may hinder the achievement of this target. The overall situation creates a vicious cycle of poverty, loss of productivity, chronic illness, disability, and social stigma (Figure 2).^13^

Nevertheless, coordinated global initiatives could and have played an important role, and examples of notable successes include the near elimination of dracunculiasis, lymphatic filariasis, and trachoma in several countries, as well as the significant reduction in human African trypanosomiasis (HAT) cases.^14^ Integrated multidisciplinary approaches that align drug discovery with sustainability principles, including green chemistry,^8^ and the creation of public–private partnerships such as the Drugs for Neglected Diseases initiative (DNDi), can promote sustainable pipelines of new therapeutic agents. Therefore, medicinal chemistry has a vital role to play in this direction, enabling the creation of innovative, affordable, and easily accessible treatments, while combating the rise of drug resistance and the spread of new disease vectors.^15^

Over the years, significant steps have been taken in this direction, and we have chosen just a few to highlight here.

**Figure 3:**
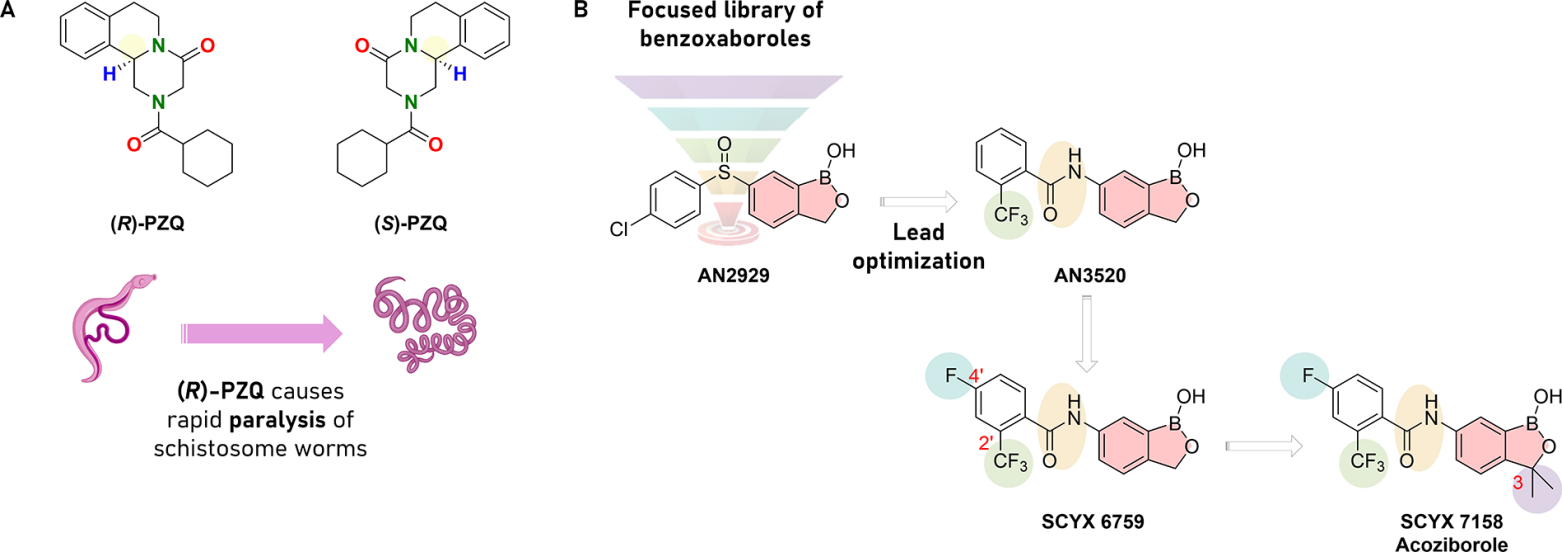
(A) Praziquantel used to treat schistosomiasis and the biological activity of the corresponding (*R*)/(*S*) enantiomers. (B) Drug discovery campaign to identify acoziborole for treating HAT.

One of the goals outlined in the SDGs is the eradication of schistosomiasis, which is endemic in 78 low- and middle-income countries and for which the current standard of care is praziquantel (PZQ, in Figure 3A). In 1972, PZQ was discovered thanks to a joint collaboration between Bayer AG and Merck KGaA. Its chemical structure is asymmetric with the two stereoisomers, which, according to the basics of medicinal chemistry, have a different antischistosomal activity (Figure 3A). Specifically, the (*R*)-PZQ is the biological active eutomer which causes rapid paralysis of schistosome worms at nanomolar concentrations and the (*S*)-PZQ is the distomer, with lower anthelmintic activity plus an unpleasant smell, bitter taste, and worse side-effects. Although this difference has been known since 1983, PZQ is still administered as a racemic mixture. To note, PZQ is only formally approved for children older than 4 years, leaving 50 million infected preschool-aged children untreated. To address this problem, efforts to produce pure (*R*)-PZQ rather than the racemic mixture for these children have been successful by reducing the tablet size, suppressing the offensive taste and odor, and reducing side-effects.^16^ Therefore, a novel orodispersible tablet of (*R*)-PZQ (arpraziquantel) has been developed and successfully passed phase 1 and 2 trials.^17^

Another disease that has been recognized as potentially impacting the achievement of the 2030 Agenda is HAT (also known as sleeping sickness), caused by different *Trypanosoma brucei* specie*s*.^14^ It is a NTD that, by affecting both people and livestock, straddles the ground between human health, livestock health, agricultural production, and rural development in Africa. The disease, fatal if left untreated, progresses through two distinct stages*:* an initial acute stage (stage 1) where the parasitic infection is restricted to the hemolymphatic system and a second stage (stage 2) where parasites have migrated across the blood–brain barrier (BBB). This latter is particularly difficult to treat because the only available drugs, melarsoprol and eflornithine, have limited ability to cross the BBB, are toxic, and their activity depends on complex parenteral administration, which is a barrier to treatment. In addition, the high level of antigenic variation makes vaccine development impractical, meaning that a safe, orally administered drug effective against both stages of HAT is urgently needed. With the aim of eliminating the need for staging and increasing the potential for eradicating sleeping sickness, Anacor Pharmaceuticals conducted a whole-cell viability assay of a library of benzoxaboroles and identified compound AN2929 with an IC_50_ of 0.12 μg/mL against *Trypanosoma brucei*.^18^ The sulfoxide compound was effective at 20 mg/kg i.p. but failed to show a complete cure when administered orally. The role of medicinal chemistry proved to be essential in overcoming this bottleneck. In fact, a focused structure–activity relationship (SAR) of the linkage groups found the oxaborole carboxamides as highly permeable and metabolically stable alternative (AN3520, Figure 3B). Pharmacokinetic analysis demonstrated that AN3520 and even more its 4′-fluorine derivative SCYX-6759 were orally bioavailable and able to cross the BBB. However, while SCYX-6759 was fully efficacious in the stage 2 model following twice-daily intraperitoneal administration at a dose of 50 mg/kg, it exhibited only partial efficacy in the same model following twice-daily oral administration. To further improve the pharmacokinetic properties of the series, the installation of substituents at the C(3) position of the benzoxaborole scaffold provided the best balance of potency and pharmacokinetic profile (Figure 3B). On this lead compound, a phase 1 clinical trial was successfully completed in 2015, and a phase 2/3 trial was initiated by the DNDi in 2016, meaning that acoziborole holds promise in the efforts to reach the WHO goal of interrupting HAT transmission by 2030.^19^

**Figure 4:**
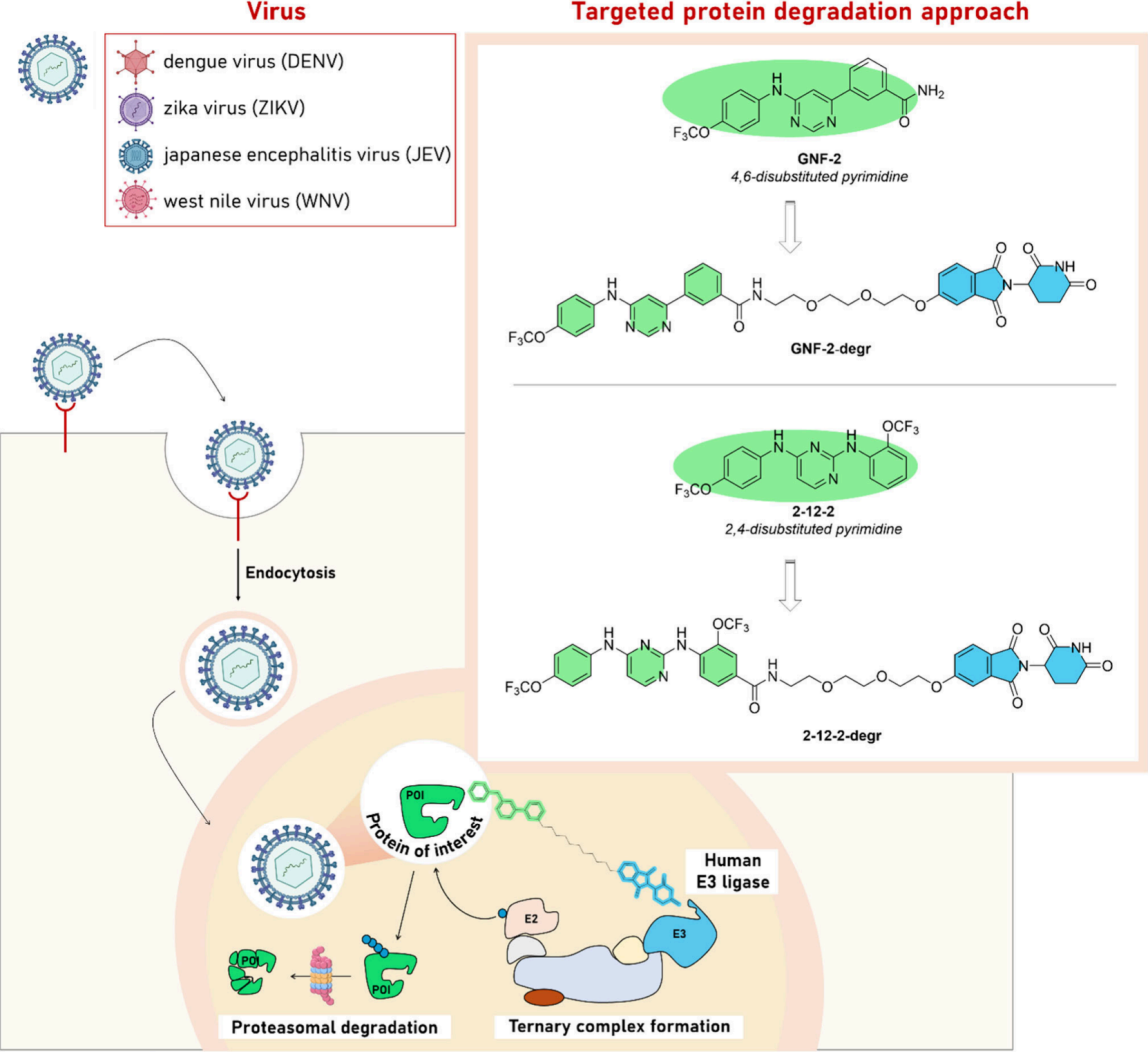
On the left, the mechanism of action of antiviral PROTAC is represented. On the right, medicinal chemistry evolution from inhibitors to broad-spectrum degraders (GNF-2-degr and 2-12-2-degr). This figure was created with BioRender.com.

Another disease where medicinal chemistry can make a difference is dengue fever, another IdoP. The WHO classifies dengue fever as one of the world’s 20 NTDs, diseases that serve as “prox(ies) for poverty and disadvantage”, and prescribes population-targeted interventions to control dengue in impoverished and marginalized communities.^14^ In a screening campaign aimed to identify small molecules inhibiting any step(s) of the dengue virus (DENV) replication cycle, Gray’s group determined that compound GNF-2 (Figure 4), an allosteric inhibitor of Abelson (ABL) kinases, showed antiviral activity not only through its known kinase inhibitory profile but also through interactions with the E protein on the virion surface.^20^ Afterward, medicinal chemistry work led to the identification of GNF-2 analogues (2,4-disubstituted diaminopyrimidines) that lost the breakpoint cluster region (BCR)-ABL kinases activity but improved the DENV profile in the viral infectivity assay (2-12-2: BCR-ABL, IC_50_ > 10 μM; DENV 1–4, IC_90_ in the range of 20–40 μM). Although these molecules provided proof-of-concept for antiviral activity, their potency did not meet the criteria required for effective antiviral drug development. At this stage, it is pleasing to see how medicinal chemistry, by applying to the IDoPs the most innovative pharmacological modality such as the Proteolysis Targeting Chimeras (PROTACs), made a big difference. In fact, the same group showed the superior activity of the degrader molecules compared to the parental inhibitors with the PROTAC molecules having EC_90_ values in the single digit micromolar value across Zika Virus, Japanese Encephalitis Virus, and West Nile Virus (WNV) Kunjin. Specifically, GNF-2 exhibits very weak activity against WNV Kunjin (EC_50_ > 20 μM), whereas GNF-2-degrader has comparable activity (EC_90_ values in the single-digit micromolar) against the mosquito-borne flaviviruses checked (Figure 4). These data illustrate that conversion of an antiviral inhibitor to a degrader can be advantageous with respect to antiviral potency as well as spectrum of activity^21,22^ and confirm that, although in its infancy and with many challenges ahead, PROTAC-mediated protein degradation can be a transformative technology for the development of next-generation NTD drugs.^23^

**SDG #3**. **Good Health and Well-Being**. *“Ensure healthy lives and promote well-being for all at all ages*.*”*^1^

In support of human health and well-being, medicinal chemistry research spans a wide range of applications. Unmet medical needs (UMNs) are, of course, the therapeutic areas that require additional attention, where therapeutic treatments are not yet available or are not satisfactory and where medicinal chemists can continue to make a difference.

**Figure 5:**
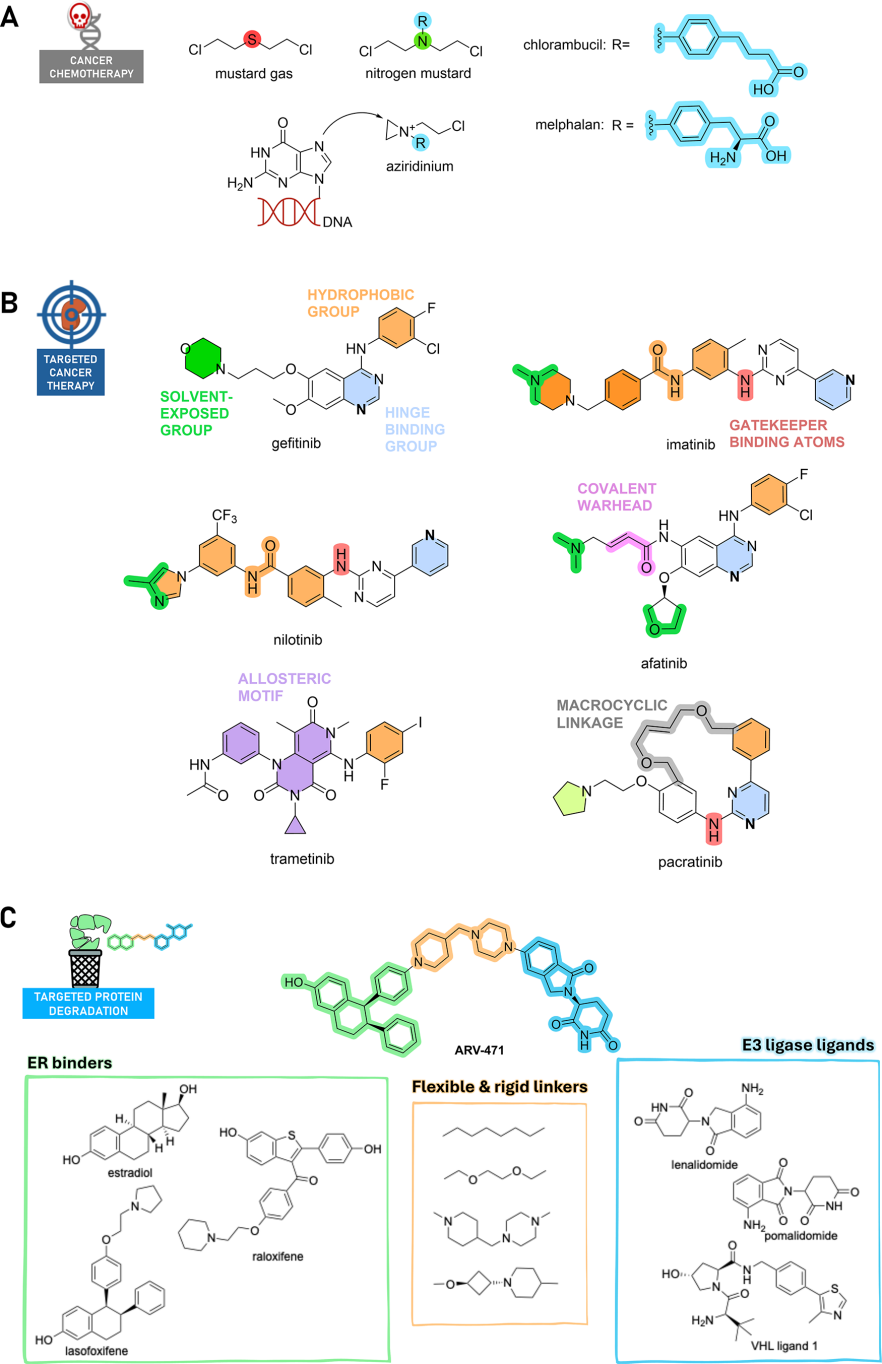
(A) Chemical structures of nitrogen mustards together with the mechanism of action. (B) Chemical structures of small molecule kinase inhibitors, whose pharmacophoric elements are highlighted in different colors. Light-blue groups bind the hinge region, and the atoms involved in hydrogen bonds are shown in bold. The green atoms are solvent-exposed, while the orange moieties interact in the hydrophobic pocket(s). The gatekeeper binding atoms are depicted in red, while the covalent warhead and the macrocyclic linkage are shown in pink and gray, respectively. The allosteric structural motif is violet. (C) Chemical structure of ARV-471 along with the exploration of key PROTAC elements.

In the landscape of cancer treatment, the contribution of medicinal chemists over more than a century is beyond dispute. By chance, research on war gases led to the discovery of cytotoxic nitrogen derivatives as the first anticancer chemotherapeutic agents.^24^ Aliphatic mustards are highly reactive, forming aziridinium ions that attack DNA and also any surrounding nucleophile, causing massive damage. Replacing aliphatic nitrogen with an aromatic one reduces reactivity through lone pair delocalization, leading to approved chemotherapeutic agents, including chlorambucil and melphalan in 1957 (Figure 5A). However, many limitations related to high toxicity and poor selectivity still exist. Hence, medicinal chemists along with advances in the field of cancer biology have made a substantial push for a paradigm shift from conventional chemotherapy to targeted cancer treatment.^25,26^ Unlike conventional chemotherapy drugs that indiscriminately kill rapidly dividing cells, targeted drugs are designed to specifically block the effects of proteins, mainly kinases, whose activity is restricted to cancer cells. Targeted drugs can thus specifically target cancer cells but spare normal cells, hence having high potency and low toxicity. The impact of targeted cancer therapy is underscored by the approval of ∼80 kinase inhibitors, with seven new ones marketed in 2023, making kinases the most important drug targets in the 21st century.^25^ Over the past 20 years, the development of kinase inhibitors has exemplified modern medicinal chemistry concepts based on structure-guided optimization approaches and new chemical modalities. We have witnessed a remarkable transformation, not only in rational design, but also in creative, disruptive approaches that have enabled novel modalities to enter the clinic.^27^

Inspired by the interactions made by the ATP with the kinase target, type I inhibitors that bind in the ATP binding pocket of active kinase represent the most exploited strategy by medicinal chemists.^28^ Typical pharmacophoric elements are those found in gefitinib (Figure 5B), an anilino-quinazoline epidermal growth factor receptor (EGFR) inhibitor:^29^ (i) the quinazoline ring is the hinge-binding moiety (highlighted in light blue in Figure 5B), which interacts in a similar manner to the purine ring of ATP; (ii) a hydrophobic group (highlighted in orange in Figure 5B), which binds in a conserved hydrophobic pocket located at the back of the active site, providing the basis for the superior affinity of these compounds over ATP; and (iii) a solvent-exposed group at position 6 (highlighted in green in Figure 5B) to improve pharmacokinetic properties. However, because the ATP-binding site is highly conserved among kinases, gefitinib has activity against several other protein kinases. Against this drawback, medicinal chemists sought to include into type-I pharmacophore an extra hydrophobic moiety (highlighted in orange in Figure 5B) directed to occupy a less conserved pocket formed by kinase inactive conformation (the so-called “DFG-out”), leading to type-2 inhibitors.^29^ Imatinib (Figure 5B), a 2-amino-4-pyrido-pyrimidine Bcr-Abl inhibitor, is the protype of this class, which represents an unprecedented medicinal chemistry success. The amino-pyrimidine-pyridine moiety is in the adenine pocket, and the other part of the drug occupies the gate area, by interacting with the gatekeeper T315 via the aniline function (colored red in Figure 5B). Despite the hype, point mutations of Bcr-Abl at gatekeeper residues (e.g., T315I) alter imatinib binding and induce conformational changes leading to drug resistance. Nilotinib (Figure 5B) has been purposely designed to overcome the T315I mutation. The structure of imatinib has been manipulated by inverting the amide linking group, by replacing the piperazine ring with 3-methylimidazole, and by adding trifluoro-methyl group to the benzene. This design strategy has led to the sequential approval of different imatinib-resistant Bcr-Abl kinase inhibitors.^29^ Apart from optimization of Type I/II inhibitors, medicinal chemists have struggled to solve these issues, further emphasizing the need to explore other mechanisms of action potentially leading to the desired kinase inhibition profile.^30^

Allosteric kinase modulators (also called type III inhibitors), which bind outside the ATP binding pocket without interacting with the hinge region, have the potential to overcome mutation-associated drug resistance and selectivity issues better than previous inhibitors.^29,31^ With respect to type I/II, ATP noncompetitive allosteric modulators are a highly heterogeneous group with different chemotypes. Trametinib is a pyrido[4,3-*d*]pyrimidine derivative (shown in violet in Figure 5B) that shares a disubstituted aniline (shown in orange in Figure 5B) already present in type I/II inhibitors. Trametinib binds in the gate area and, by exploiting binding to this site adjacent to the ATP-binding pocket and specific for MEK1/2, exhibits high selectivity and activity against resistant cancers.

Additionally, covalent inhibition strategies have received renewed interest in recent years, as these can drive both potency and selectivity.^32^ The irreversible mechanism of action allows permanent disablement of kinase activity, which can only be restored upon the expression of new proteins. Medicinal chemists have rationally designed covalent inhibitors by strategically inserting an electrophilic warhead (e.g., Michael acceptors, nitriles, epoxides, aldehydes, and haloacetamides) into the structure of the reversible kinase inhibitor so that the warhead is placed close to the target residue (usually a cysteine) and can form the covalent bond. Of course, the identification of an appropriate target residue that is solvent-accessible (preferably on the surface), poorly conserved in similar kinases, and close to a druggable pocket is critical.^32^ Afatinib is an anilino-quinazoline inhibitor which bears a crotonamide as Michael acceptor site (colored pink in Figure 5B). The quinazoline ring is located in the adenine pocket, and the 3-chloro-4-fluoroanilino group is surrounded by the side chains of residues K745 and L788 in the hydrophobic back pocket. The Michael acceptor allows it to bind covalently to C797, leading to irreversible inhibition, which provides the ability to treat patients harboring EGFR-mutations resistant to gefitinib.

Novel medicinal chemistry strategies along with the synthetic chemistry advances have enriched the small molecule toolkit for targeting the kinome, providing the basis for the development of macrocyclic kinase inhibitors, as exemplified by pacratinib (Figure 5B).^33^ Medicinal chemists have rationally designed macrocyclic inhibitors by typically restricting the conformation of acyclic kinase inhibitors to improve their potency and selectivity, thanks to their limited conformational freedom and their well-defined 3D shape. Moreover, macrocyclization may improve the pharmacokinetic profile including cell permeability and oral bioavailability.^33^ Pacratinib has been designed by connecting the open ends of an anilinopyrimidine hit, so that the binding to the hinge region can be maintained and the macrocyclic linkage (highlighted in gray in Figure 5B) easily formed by exploiting the ring-closing metathesis reaction. Pacratinib, bearing benzylic ether oxygens connected via a but-2-ene spacer, turned out to be not only a potent inhibitor of wild-type and mutant JAK2 but also highly selective compared to other JAK2 clinical candidates.

Unfortunately, patients often discontinue treatment with kinase inhibitors due to toxicity, resistance, or disease progression. As a result, other modalities have been suggested by medicinal chemists to potentially address the limitations seen with kinase inhibitors. PROTAC technology has showcased a significant impact globally by improving human health and well-being, especially for UMN, such as metastatic breast cancer. Developed by Arvinas and Pfizer, ARV-471 (Figure 5C) is a PROTAC degrader for the estrogen receptor (ER), which received the fast-track designation from the FDA in 2024 for the treatment of ER^+^/HER2^–^ metastatic breast cancer, insensitive to endocrine-based therapy. However, its development was not similarly fast and took approximately 15 years. At the infancy of PROTAC technology, only a few peptide binders were available to recruit the E3 ubiquitin ligase. The first ER-directed PROTACs were developed by using estradiol as ER binder linked via an alkyl linker to an ubiquitin ligase phosphopeptide binder,^34^ which resulted in cells being impermeable and susceptible to phosphatases. To avoid this problem, the phosphopeptide was replaced with a pentapeptide,^35^ leading to cell permeable PROTACs, but it was not potent enough for *in vivo* applications.

With new nonpeptidic E3 ligase binders identified, systematic SAR studies were performed by Arvinas. Particularly, they employed raloxifene and lasofoxifene as ER binders and Von Hippel-Lindau (VHL) and thalidomide analogues as E3 ligase ligands. They also evaluated linkers of different natures with a particular focus on rigid linkers, which can significantly improve PROTAC degradation efficiency. After iterative cycles, ARV-471 was prioritized as a degrader effective against resistant-ER mutants. Notably, ARV-471 carries a piperidine-piperazine linker which, in addition to rigidity, has demonstrated the potential to improve the oral bioavailability of PROTACs by providing a protonation site.^36^ This PK property is crucial for ensuring future PROTAC accessibility to all of the global population, even in resource-poor settings.

Another recent success of medicinal chemistry in the sense of structure-based design, iterative synthesis, and analogue testing is the development of inhibitors of KRAS, mutated in up to 30% of all human cancers. Notably, over the past decade, this protein has gone from being deemed “undruggable” to yielding two clinically approved drugs and several additional candidates in the preclinical and clinical development stages.^37^

SDG #3 highlights the need to ensure healthy lives for all at all ages, with a special focus on elderly people in our aging society. Importantly, the PROTAC technology has also been applied to other highly UMN, such as neurodegenerative diseases. ARV-102 (structure undisclosed) is an investigational oral PROTAC degrader uniquely designed to cross the BBB and degrade leucine-rich repeat kinase 2 (LRRK2) protein.^38^

LRRK2 is a validated target for devastating diseases such as Parkinson’s disease and progressive supranuclear palsy, characterized by increased activity, expression, or mutations of LRRK2. However, being a large multidomain scaffolding kinase, LRRK2 is considered a difficult-to-target kinase,^39^ making the development of classical kinase inhibitors highly challenging. Also in this case, PROTACs may be the winning solution. In preclinical studies, ARV-102 has been shown to cross the BBB and degrade effectively LRRK2 by nearly 90%.^38^ Now ARV-102 is in a Phase I clinical trial as the first oral PROTAC protein degrader to treat neurodegenerative diseases, marking a significant milestone for transformative therapies for patients living with these devastating diseases. Additionally, tau degraders are being developed by Arvinas and others^40,41^ to tackle currently uncurable tauopathies.

Another area of intervention for medicinal chemists contributing to SDG #3 is that of nutraceuticals, i.e., food-derived products with health-promoting or disease-preventing effects.^42^ As most of the global population continues to live longer, the incidence of chronic age-related diseases, such as cardiovascular and neurodegenerative diseases, diabetes, and cancer (e.g., gastrointestinal cancers), which are known to be related to lifestyle and dietary habits, continues to increase. As a result, nutraceuticals are currently nonmedicinal health-promoting products that are used by a large part of the population.^42^ Many efforts have been aimed at assessing their role in modifying and maintaining normal physiological function and improve well-being. As an example, the potential role of caffeine in preventing Alzheimer’s disease (AD), which is likely due to the cerebroprotective effects of adenosine A2A receptor antagonism,^43^ has led to a Phase 3 clinical trial to test caffeine’s efficacy to slow cognitive decline in early moderate AD (NCT04570085). By blocking A2A receptors (usually dysregulated in aging, AD, and tauopathies),^43^ it triggers neuronal molecular mechanisms associated with plasticity and restores memory performance, also confirmed by experimental studies on amyloid and tau animal models of AD.^44^ This research could reshape our understanding of nutraceuticals’ role in preventing and supporting well-being and health.

However, important issues associated with nutraceuticals exist for medicinal chemists. These are related to (i) the complex chemical compositions and (ii) the multiple mechanisms of action. (i) Extraction and chemical characterization can be highly challenging, as well as (ii) the *in vitro* and *in vivo* bioactivity profiling.^42^ This is exemplified by the controversial story of curcumin, which has been shown to possess physicochemical properties of implausible clinical leads.^45^ Curcumin is characterized by instability, reactivity, and poor bioavailability and has been identified as having attributes of both pan-assay interference compounds (PAINS)^46^ and invalid metabolic panacea compounds.^45^ Despite these significant issues, it is evident that natural products have historically served as a significant source of pharmaceuticals, and this trend is anticipated to continue into the future.^47^ Considering the lives that could be improved by their use, the genius of medicinal chemists can indeed help to overcome the current challenges and to redefine the concept of nutraceuticals, taking into account the efficacy, safety, and toxicity of these products, backed up by high-quality scientific evidence.

**SDG #4**. **Quality Education & SDG #10**. **Reduced Inequalities**. *“Ensure inclusive and equitable quality education and promote lifelong learning opportunities for all.” & “Reduce inequality within and among countries*.*”*^1^

Education is a cornerstone of sustainable development, especially in fields such as medicinal chemistry that are critical for driving innovation and improving human health. SDG #4 underscores the importance of inclusive, equitable, and quality education, which is essential for nurturing the next generation of scientists all over the world equipped to tackle global health challenges.^48^ In current medicinal chemistry, promoting quality education is not only about developing technical expertise but also about embedding sustainability principles into research and development.^49^ To achieve this, educational institutions must adapt their curricula to incorporate sustainable practices, such as green chemistry and ethical drug design, encouraging students to consider both the environmental and societal impacts of their work.^50^ The increasing prominence of green chemistry organizations in the USA, Europe, and UK reflects a growing recognition of the value of integrating sustainable practices into research and education. Many of these organizations prioritize education alongside research, understanding that the future of the field, and the pharmaceutical industry as a whole, depends on educating future generations. For instance, the American Chemical Society’s Green Chemistry Institute Pharmaceutical Roundtable, established in 2005 with the vision of encouraging the integration of green chemistry principles into the pharmaceutical industry, created a dedicated medicinal chemistry subgroup in 2011. This subgroup has made freely available tools specifically dealing with tips and tricks for medicinal chemists (https://acsgcipr.org/tools/medchem-tips-and-tricks/). A similar awareness applies to universities, whose mission is to discover, preserve, and disseminate knowledge and to educate the next generation of citizens. An International Master in Sustainable Drug Discovery (S-DISCO) has been created by a consortium of 4 universities, led by the University of Ghent, with the aim of providing training in drug discovery, while communicating the importance and impact of sustainability in drug discovery within local and global health systems.^51^ Among others, the MSc in Sustainable Chemistry by the University College London has been specifically developed in response to the need of pharmaceutical (and chemical) industries for professionals with interdisciplinary knowledge of the core concepts and aspects of chemical sustainability and green chemistry.^52^

Yet, initiatives like the DNDi provide real-world examples where public–private partnerships focus on developing affordable treatments for diseases like leishmaniasis or sleeping sickness while also offering opportunities for young scientists to engage in sustainable drug discovery. Additionally, programs like the WHO’s Special Program for Research and Training in Tropical Diseases,^53^ which focuses on capacity building in disease-endemic regions (including drug discovery), show how educational initiatives can strengthen the local workforce.

By equipping students with both the technical skills and the sustainable practices necessary for ethical drug development, we can help ensure that future medicinal chemists are prepared to contribute meaningfully to the 2030 Agenda. Providing quality education, particularly in those areas where often fewer career options can be found, undoubtedly enables upward socioeconomic mobility and contributes to reducing inequalities (SDG #10). As clearly highlighted by Paul Anastas, the chemistry of sustainability cannot simply be the chemistry of the rich and powerful few, as any vision of sustainable chemistry must be inextricably linked to equity.^2^ The basis for innovation for a sustainable future is to stimulate curiosity and interest in science; as such, the education is key not only to individual personal success but also to have a sustainable society. It is crucial to foster educational justice, employability, and economic participation for all people. Community engagement is an important cornerstone of social cohesion. These principles have recently inspired University College London – School of Pharmacy and Marmara University in Turkey to create the 3DI Centre, a digital virtual reality institute that purposely aims to address global inequalities in access to chemical science education and training.^54^ Using cutting-edge virtual reality technologies, the project creates immersive and inclusive environments for learning and collaboration where students and scientists can engage in hands-on training, collaborative meetings, and conferences. Educational platforms such as the drug discovery unit at the University of Dundee,^55^ which offers online courses in medicinal chemistry, foster global access to knowledge and skill development, especially for students in regions with limited educational resources. These platforms enable broader participation in the field, equipping learners with cutting-edge methodologies and sustainable practices that are essential for modern drug discovery. To note, these as well as all “virtual” initiatives not only promote educational equity by enabling students from remote and underprivileged regions to access high-quality medicinal chemistry training but also support environmental sustainability by reducing the need for travel.

**SDG #5**. **Gender equality**. *“Achieve gender equality and empower all women and girls.”*(1)

In 2017, by considering multiple parameters such as membership in professional organizations, corresponding authorship of scientific papers, and representation in professional and leadership positions, the percentage of women participating in professional medicinal chemistry activities was estimated to be less than 20%.^56^ This seemed counterintuitive given the nature of the discipline, which is inherently diverse, thrives on collaboration, and brings together disparate scientific fields.

Despite years of progress, although with different nuances, gender equality in medicinal chemistry still seems to be a goal to be achieved. Recent statistics in pharma show that women are under-represented and a gender diversity problem persists. Bibliometric analyses of publications from 23 of the biggest pharma companies suggest that, overall, there has been little change in gender balance over an 18-year period.^57^ However, there are signs of change, thanks also to several important initiatives specifically aimed at making progress toward gender equality pursued by different bodies and institutions all over the world. From the initiatives illustrated below, it appears that they are trying their best, and it is gratifying to see the commitment that they have brought to the cause.

With the aim of supporting systemic changes needed to ensure women have the opportunity to thrive across every stage of their career, several Journals have dedicated special issues to the “Women in Medicinal Chemistry” topic. *Journal of Medicinal Chemistry* and *ACS Medicinal Chemistry Letters* were the first ones,^58,59^ followed by *Bioorganic and Medicinal Chemistry* and many others.

It is also encouraging to see that many scientific organizations are working in this direction and filling the gap by writing opinion pieces^60^ and organizing special scientific meetings to highlight the careers and raise the visibility of outstanding early-career women medicinal chemists. After the first “Rising Stars: Women in Medicinal Chemistry” session at ACS Fall National Meeting in 2019,^61^ the European Federation of Medicinal Chemistry and Chemical Biology (EFMC) had its own session in 2024 at EFMC-ISMC 2024 in Rome.

Much has been accomplished, but there is still much to be done. To remain vigilant on this issue and to promote a sense of belonging, relevance, and empowerment, the women of the ACS Medicinal Chemistry Division (MEDI) recently shared uplifting stories of what inspired them to become medicinal chemists. For the past 2 years, ACS MEDI has published an editorial on March 8th (International Women’s Day) encouraging other women in the field.^61,62^

Another issue that should be considered as integral to achieving gender equality in Medicinal Chemistry is to acknowledge that women’s health research and development has been neglected over the years, standing as a therapeutic area that requires additional attention, where current therapeutic tools are not yet available or satisfactory. A timely editorial by Wendy Young is a call to action for medicinal chemists to recognize and embrace the growing responsibilities and the largely untapped research opportunities.^63^

**SDG #7**. **Affordable and Clean Energy**, **SDG #9**. **Industry**, **Innovation**, **and Infrastructure, & SDG #13**. **Climate Action**. *“Ensure access to affordable*, *reliable*, *sustainable and modern energy for all*.*”*, *“Build resilient infrastructure*, *promote inclusive and sustainable industrialization and foster innovation*.*”, & “Take urgent action to combat climate change and its impacts*.*”*^1^

Sustainability in drug discovery is increasingly recognized as a critical component in ensuring long-term access to lifesaving treatments while minimizing the environmental impact of pharmaceutical operations. However, the sector faces significant challenges, particularly in reducing carbon dioxide (CO_2_) and greenhouse gas (GHG) emissions, meeting recycling targets, and improving air quality. Despite not being classified as a heavy industrial sector, the pharmaceutical industry significantly impacts environmental pollution. A report from the UK’s National Health Service in 2021 highlighted that medicines account for about a quarter of its carbon footprint, underscoring the urgent need for greener practices in drug manufacturing.^64^ With the continued depletion of fossil fuels and the accelerating impact of climate change, the pharmaceutical sector must take decisive action to lower its emissions.

Leading pharmaceuticals companies, such as Pfizer, AstraZeneca, and Merck, are already taking steps to align their operations with SDG #7, #9, and #13, committed to reduce GHG emissions, to carbon neutrality, and to net zero emissions between 2025 and 2050. These companies are working to reduce energy consumption, water use, waste, and pollution in every stage of drug development while opting for the use of renewable energy and resources. In this section, we decided to report a few representative examples, although this is not an exhaustive list.

Pfizer, for instance, began its commitment to green chemistry in the early 2000s, with its 2002 U.S. Presidential Green Chemistry Award for developing a greener synthetic route for sertraline (Zoloft), which revolutionized the production process of one of the most prescribed antidepressants.^65^ Then in 2008, Pfizer developed the first Reagent Selection Guide,^66^ pioneering advancements in green and sustainable chemistry. Over the years, Pfizer has expanded its sustainability efforts, culminating in a 2022 commitment to achieve net-zero emissions by 2040, a decade ahead of most industry targets.

Similarly, AstraZeneca’s “Ambition Zero Carbon” initiative sets ambitious science-based decarbonization goals, including reducing absolute energy use, doubling energy productivity by 2025, and using 100% renewable energy for its operations. AstraZeneca’s research into sustainable drug discovery also includes innovations like late-stage functionalization (LSF),^67^ which streamlines drug synthesis by reducing resource-intensive reaction steps, thereby generating molecular diversity more quickly and more sustainably.

As with other pharmaceutical companies, Merck has established a strong track record of delivering Corporate Sustainability goals. Building on the SDGs, Merck is committed to three main goals: achieving human progress for more than one billion people through sustainable sciences and practices and integrating sustainability into all their value chains by 2030, as well as achieving climate neutrality by 2040. Merck’s development of tools like the industry-first quantitative tool DOZN,^68^ a unique web-based greener alternative scoring matrix, which helps assess the sustainability of chemical processes, and the SMART-PMI (in-Silico MSD Aspirational ResearchTool),^69^ which predicts process mass intensity (PMI) from molecular structure, exemplifies its commitment to improving sustainability in pharmaceutical production. Merck has also pioneered the use of flow chemistry, which improves the scalability, reproducibility, and efficiency of reactions, reducing waste and environmental impact. Importantly, flow chemistry was included among the first selection of the Top Ten Emerging Technologies in Chemistry,^70^ released in 2019 as a special initiative held by the International Union of Pure and Applied Chemistry (IUPAC) honoring its 100th anniversary. Through application of flow chemistry, Merck chemists achieved the flow synthesis of ciprofloxacin,^71^ an antibiotic in the Essential Medicine list.

These examples from leading companies demonstrate that the pharmaceutical industry can significantly contribute to SDGs #7, #9, and #13, while ensuring access to affordable, effective medicines for global populations.

**SDG #12**. **Responsible Consumption and Production**. *“Ensure sustainable consumption and production patterns*.*”*^1^

Sustainable practices are crucial in addressing environmental challenges caused by industrialization. The critical issue lies in material extraction and end-of-life waste accumulation, which depletes natural resources and degrades ecosystems.^72^ Biowaste residues from food, agriculture, and forestry industries is a significant global problem, generating billions of tons annually. Traditional disposal methods such as burning and landfilling release harmful pollutants and GHG. However, innovative approaches in medicinal chemistry might offer a promising solution. By transforming low-value biowaste into valuable products such as biologically active compounds, medicinal chemists can convert agricultural and food waste into high-value molecules, thus reducing environmental impact while supporting a circular economy based on renewable feedstocks instead of fossil-based resources.^73^ In this respect, the potential for producing bioactive compounds from agricultural and food waste is, in principle, enormous. These materials, rich in valuable chemical components, might provide a nearly inexhaustible source of high-value molecules.

**Figure 6:**
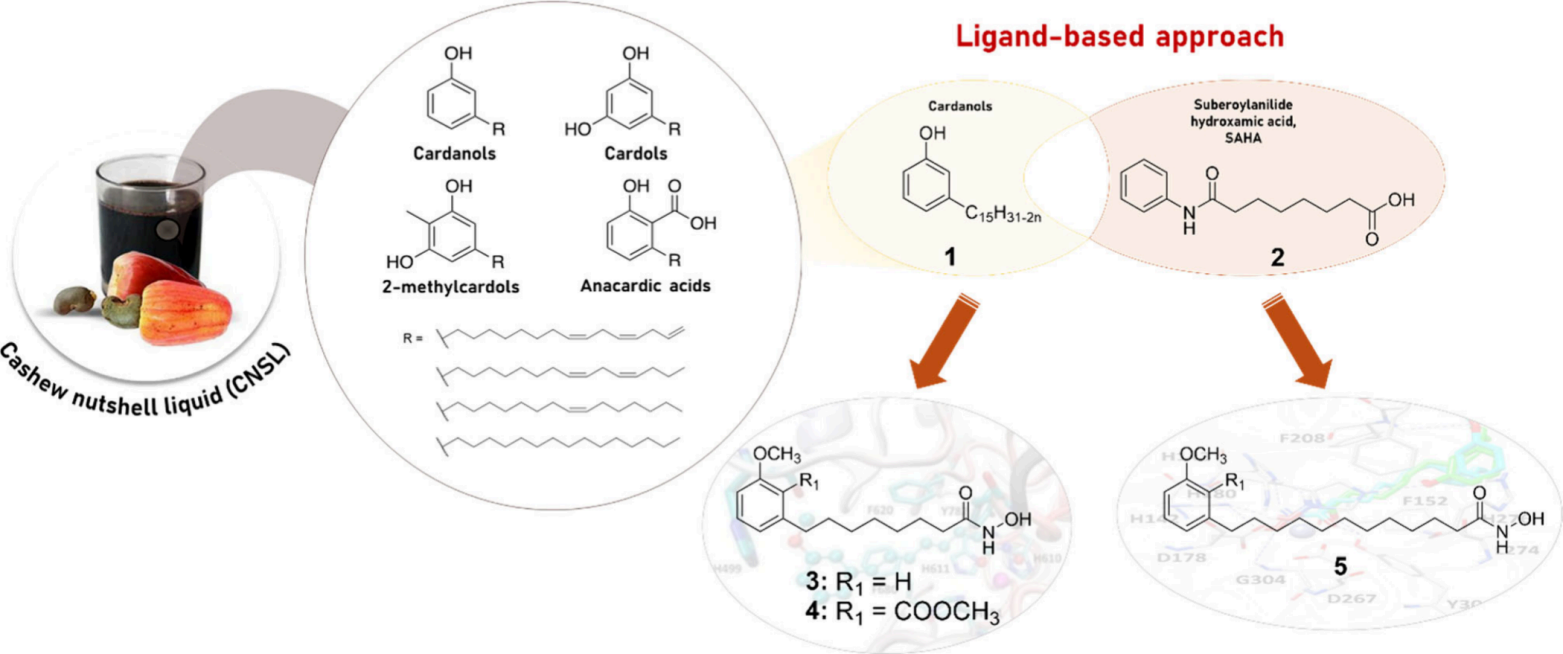
Antiparasitic compounds **3**–**5** obtained from CNSL, a byproduct of the cashew nut industry.

One example of medicinal chemistry endeavors based on agroindustrial waste valorization is the use of cashew nutshell liquid (CNSL) to develop new drugs. CNSL, a byproduct of the cashew nut industry, is rich in phenolic compounds, including anacardic acid, cardanol, cardol, and 2-methylcardol (Figure 6), which exhibit several bioactive properties, such as antimicrobial, antioxidant, and anti-inflammatory effects. While these compounds are not yet potent enough to serve as standalone drug candidates, CNSL is a promising starting material for medicinal chemistry. However, only a few examples of converting CNSL into pharmaceuticals have been reported,^74,75^ despite the fact that CNSL offers a compelling opportunity for biobased drug manufacturing in the regions where it is produced, particularly in Asia and Africa. These areas, being major CNSL producers, could ideally establish local pharmaceutical industries that address public health needs while stimulating economic growth, creating jobs, reducing pharmaceutical import dependence, and minimizing carbon emissions from long-distance transportation. By integrating drug production with waste valorization in endemic regions (SDG #12), medicinal chemists can create a win-win scenario: developing affordable, biobased medicines (SDG #3) while supporting sustainable, locally driven economic growth (SDG #8).^15^

Inspired by these concepts and employing a ligand-based approach, our group, in 2019, leveraged the chemical similarity between cardanols (**1**) and the capping group of suberoylanilide hydroxamic acid (SAHA, **2** in Figure 6) to design and synthesize the first sustainable-by-design histone deacetylase (HDAC) inhibitors, **3** and **4**, derived from CNSL. These compounds exhibited therapeutic potential comparable to the investigational AD drug SAHA (Figure 6).^76^ More recently, in line with this strategy and incorporating greener methods, we further developed a small library of CNSL-based antiparasitic compounds obtained through a green metathesis approach (Figure 6). Among them, derivative **5** (Figure 6) was identified as an interesting hit compound toward *T*. *b*. *brucei* responsible for African animal trypanosomiasis.^77^

**SDG #14**. **Life Below Water & SDG #15**. **Life on Land**. *“Conserve and sustainably use the oceans*, *seas and marine resources for sustainable development*.*” & “Protect*, *restore and promote sustainable use of terrestrial ecosystems*, *sustainably manage forests*, *combat desertification*, *and halt and reverse land degradation and halt biodiversity loss*.*”*^1^

SDG #14 and SDG #15 are interdependent goals within the broader sustainability framework, both focusing on the conservation and sustainable use of ecosystems (marine and terrestrial) essential to maintaining global biodiversity, with disruptions to one often affecting the other.^78^ Pharmaceuticals and their byproducts, which persist in aquatic environments and migrate to terrestrial ecosystems, can disrupt reproductive processes in marine organisms and degrade land biodiversity.^79^ This issue is exacerbated by the increasing global consumption of pharmaceuticals, which reached 4.5 trillion doses in 2020, as drugs and their metabolites, additives, and excipients accumulate in ecosystems, often at concentrations that pose significant ecological risks.^80^ As a result, pharmaceuticals, which have been credited with saving millions of lives, have emerged as a new class of pseudopersistent organic pollutants, also known as “*pharmaceutically active micropollutants*” in aquatic environments, with concentrations in water matrices increasing dramatically over time from the ng/L to the μg/L range.^81^

To meet the targets of SDG #14 and SDG #15 by 2030, a comprehensive and multifaceted approach is needed. This includes strengthening regulatory frameworks for the discharge of pharmaceutical contaminants, revising current discharge regulations, and advancing technologies to effectively remove these pollutants from water. At the same time, a crucial aspect of mitigating environmental damage from pharmaceuticals is addressing the lifecycle of drugs from development to disposal. Medicinal chemists have a crucial role in mitigating the environmental impacts of pharmaceuticals. By incorporating sustainability into drug design and adopting “*benign by design*” principles,^82^ they can ensure that drugs do not persist in the environment, minimizing ecological harm and supporting both SDG #14 and SDG #15. Sustainable molecular design is a critical tool in the fight to protect both marine and terrestrial ecosystems. By incorporating green toxicology into the drug discovery process,^83^ medicinal chemists can identify and mitigate potential ecotoxicity early on. By analyzing the specific case of R&D and drugs for parasitic vector-borne diseases and their environmental impacts, De Koning et al. emphasize the lack of ecotoxicological testing and call for sustainable drug development practices avoiding typical contamination risks to ecosystems and nontarget organisms. These practices should include the One Health approach to drug discovery, which considers the interconnectedness of human, animal, and environmental health.^84^

**Figure 7:**
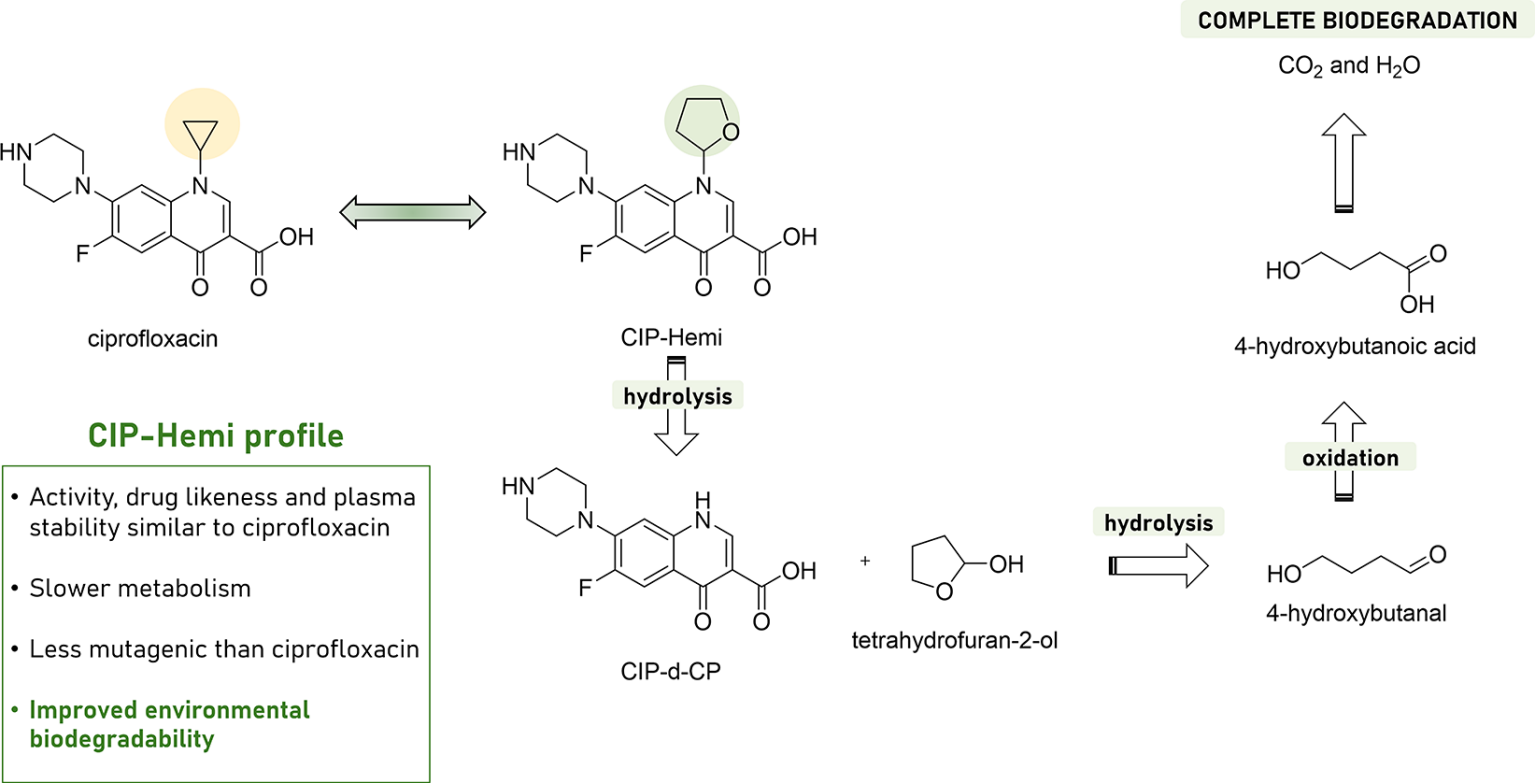
Structure, properties, and environmental biodegradation of CIP-Hemi.

A thorough analysis of the critical features responsible for ecotoxicity, along with strategies to mitigate these effects, is essential.^85^ Given the time-consuming and costly nature of generating extensive experimental data, which often requires the use of numerous animals, computational modeling or *in silico* approaches have emerged as efficient tools for risk assessment, risk management, and filling data gaps. Existing benign-by-design workflows facilitate the integration of sustainable molecular design into the drug development process.^86^ A successful application of this approach is represented by the development of an antibiotic with improved environmental properties by Kümmerer and collaborators.^87^ Starting from highly persistent ciprofloxacin (CIP), CIP-Hemi was designed to maintain its required metabolic stability and antibiotic activity against the target while being transformed into an inactive fragment (CIP-d-CP) and a linker degradable under acidic conditions, e.g., when released into the environment (Figure 7). As highlighted in this work, this innovative methodology aligns with the emerging field of soft drug development,^88^ which focuses on creating safer drugs by considering their metabolic processes during the design phase. Interestingly, this approach has already led to the development of several successful pharmaceuticals, including esmolol, remifentanil, and loteprednol etabonate,^89^ that are metabolized into inactive compounds after achieving their therapeutic effects.

In the broader context of biodiversity preservation, which is crucial for both SDG #14 and SDG #15, medicinal chemists might also play a role. As highlighted in a recent perspective by Cernak and collaborators, preserving biodiversity is not only in our self-interest as human beings but should be also a goal for the field of medicinal chemistry.^90^ The conservation of biodiversity is closely linked to drug discovery as many medicines are derived from wild species. As we face escalating challenges such as habitat loss and climate change, the role of medicinal chemists becomes increasingly vital in preserving biodiversity.^90^ While foresters and veterinarians typically lead conservation efforts, medicinal chemists could bring essential expertise to the table, particularly in conservation medicine. By being engaged in these efforts, medicinal chemists can use their expertise to directly address diseases that threaten species survival, preventing the extinction of endangered species. Moreover, understanding wildlife disease plays a crucial role in the One Health approach, serving as key strategy for preventing future pandemics.^90^ Remarkably, several pharmaceutical companies, including Novartis, Bayer, GSK, and Sanofi, started to incorporate biodiversity conservation into their sustainability strategies, followed by many others over the years. Tools like the WWF Biodiversity Risk Filter^91^ help these companies assess their environmental impact, while ensuring that their operations contribute positively to both biodiversity preservation and the future of drug discovery. By advancing sustainable drug development, strengthening regulatory frameworks, and fostering industry responsibility, we can protect both marine and terrestrial ecosystems, ensuring a sustainable future for biodiversity and human health.

**Zero Hunger (SDG #2) & Clean Water and Sanitation (SDG #6)**. “*End hunger*, *achieve food security and improved nutrition and promote sustainable agriculture*” *&* “*Ensure availability and sustainable management of water and sanitation for all*”.^1^

As the global population is expected to exceed 9 billion by 2050, to eradicate hunger and malnutrition globally, agricultural practices must evolve to meet rising food demands without compromising the environment. Indeed, although agrochemicals can significantly increase crop yields, environmental risks remain. Pesticides and fungicides often share biological pathways with plants, humans, and animals. This poses potential risks of toxicity, underscoring the necessity for sustainable practices that prioritize environmental and human safety.^80,92^ At the same time, because of these clear links and the many commonalities between medicinal chemistry and agriculture, the two sectors should join forces and share best practices for a win–win interaction.^93^ This will have clear benefits in terms of clean technology for the environment^93^ and in the development of better agrochemical fungicides and pharmaceutical antifungals.^94^

Sustainable development also requires addressing the interconnected challenges of nutritional security and clean water, as food, sanitation, and potable water systems are deeply interrelated, forming the foundation of public health. This interdependence aligns with the One Water/One Health paradigm, emphasizing the need to recognize these connections and guide action. Ultimately, achieving SDG #2 and SDG #6 requires an integrated approach that recognizes the interdependence of clean water, food security, and health, where medicinal chemistry may do its part. By applying green chemistry principles, advancing sustainable drug discovery practices, and fostering collaboration across disciplines, medicinal chemists can contribute significantly to these global goals, ensuring a healthier and more sustainable future for all.

**Decent Work and Economic Growth (SDG #8) & Sustainable Cities and Communities (SDG #11)**. *“Promote sustained*, *inclusive and sustainable economic growth*, *full and productive employment and decent work for all” & “Make cities and human settlements inclusive*, *safe*, *resilient and sustainable”*.^1^

Reducing pollution and improving public health through medicinal chemistry can, in turn, make urban areas more sustainable and livable. The fight against “noninclusive” and “unsustainable” societies is daunting, but advances in scientific tools and strategies are making waves. Medicinal chemists, like all other scientists, can contribute to solutions for improving dignity at work and economic development, along with sustainable cities and communities, especially in low-income countries, by pioneering new, holistic approaches. These should combine transfer skills, which is fundamental to promoting sustainable economic development, together with many other capacity building activities. As illustrated by G. Costantino at the EFMC-ISMC 2024^95^ in a round table discussion dedicated to the pharmaceutical sector in sub-Saharan Africa, the path to sustainable and inclusive socio-economic development could include the creation of academic spin-offs, such as B.ethical,^96^ which aims to valorize and promote the local and traditional knowledge of Central East African regions by expanding the use of their natural ingredients in Europe. This will allow us to ensure a fair profit for local workers and to develop an internal entrepreneurial spirit.

**SDG #16**. **Peace**, **Justice**, **and Strong Institutions & SDG #17**. **Partnership for the Goals**. *“Promote peaceful and inclusive societies for sustainable development*, *provide access to justice for all and build effective*, *accountable and inclusive institutions at all levels” & “Strengthen the means of implementation and revitalize the global partnership for sustainable development”*.^1^

Because of the inherently multidisciplinary nature of medicinal chemistry and the collaborative nature of drug discovery and development, “Partnership for the Goals” should be the motto for medicinal chemists. As a step forward, it would be nice if collaborative efforts and partnership could go hand in hand with science diplomacy endeavors. This has been done, but it should be done with more vigor at this sensitive time when the world is facing the worst war scenario in half a century, with wars in Ukraine and the Middle East. A successful example is the Malta conferences, which use science diplomacy as a bridge to peace in the Middle East. Scientists (including medicinal chemists) from countries or regions whose governments are hostile to each other participate in workshops with Nobel Laureates to seek solutions to problems beyond geopolitics that face this part of the world.^97^ More conferences similar to the Malta one or dedicated sessions at the big medicinal chemistry meetings should be organized, especially in those regions that are currently experiencing conflicts.

## Conclusions

We live in a rapidly changing world. Although the function of science has always been to help mankind to understand the natural world and to improve our lives using this knowledge and understanding, there was a time when chemists were perceived as smart, hardworking, eccentric men, living isolated in their laboratories and being detached from their society. Fortunately, this stereotype that has grown over time through literature and cinema is long past, with the idea of isolated male scientists working alone becoming anachronistic and surely unproductive. For medicinal chemistry, it is essential to integrate the knowledge and skills from different disciplines and sit at tables where different scientific languages are spoken. Today, medicinal chemistry should not only face the already complex endeavor of finding a new drug but also face the complex challenges posed by the SDGs. Now that we are beyond the halfway mark to achieving the 2030 Agenda for SDGs, the UN points out that scientists can solve immediate practical problems while addressing long-term goals. It is time for us, as medicinal chemists with the potential to contribute to many of the SDGs, to turn words into deeds and statements into actions and do our part. We see an exciting future for medicinal chemistry as we move toward Agenda 2030, and we believe that the advances of the next few years will far exceed those of the past decade.

A career in medicinal chemistry is fascinating, but if you approach and live it with the conviction that you can contribute to the SDGs, then it is a dream.

## Abbreviations

ABL: Abelson
AD: Alzheimer’s disease
BBB: blood–brain barrier
BCR: breakpoint cluster region protein
CO_2_: carbon dioxide
CIP: ciprofloxacin
CNSL: cashew nutshell liquid
DNDi: Drugs for Neglected Diseases initiative
DENV: dengue virus
EFMC: European Federation of Medicinal Chemistry and Chemical Biology
EGFR: epidermal growth factor receptor
GHG: greenhouse gas
HAT: human African trypanosomiasis
HDAC: histone deacetylase
IDoPs: Infectious Diseases of Poverty
IUPAC: International Union of Pure and Applied Chemistry
LRRK2: leucine-rich repeat kinase 2
LSF: late-stage functionalization
NTDs: Neglected Tropical Diseases
PAINS: pan-assay interference compounds
PMI: process mass intensity
PROTACs: Proteolysis Targeting Chimeras
PZQ: Praziquantel
SAR: structure–activity relationship
SDGs: sustainable development goals
SAHA: suberoylanilide hydroxamic acid
UN: United Nations
UNM: unmet medical needs
VHL: Von Hippel-Lindau

## References

[ref1] UN. Transforming our world: the 2030 Agenda for Sustainable Development. (UN, New York, 2015). https://sdgs.un.org/2030agenda (accessed 2024-06-14).

[ref2] Anastas P. T.; Zimmerman J. B. The United Nations Sustainability Goals: How Can Sustainable Chemistry Contribute?. Curr. Opin. Green Sustain. Chem. 2018, 13, 150–153. 10.1016/j.cogsc.2018.04.017.

[ref3] Axon S.; James D. The UN Sustainable Development Goals: How Can Sustainable Chemistry Contribute? A View from the Chemical Industry. Curr. Opin. Green Sustain. Chem. 2018, 13, 140–145. 10.1016/j.cogsc.2018.04.010.

[ref4] Cole-Hamilton D. The Role of Chemists and Chemical Engineers in a Sustainable World. Chem. - A Eur. J. 2020, 26 (9), 1894–1899. 10.1002/chem.201905748. 32003497

[ref5] da S.M. Forezi Luana; Ferreira Patricia G.; de Carvalho Alcione S.; de C. da Silva Fernando; Ferreira V. F. Medicinal Chemistry for Sustainable Development. Curr. Top. Med. Chem. 2023, 23 (11), 957–969. 10.2174/1568026623666230517114621. 37198981

[ref6] Wynendaele E.; Furman C.; Wielgomas B.; Larsson P.; Hak E.; Block T.; Van Calenbergh S.; Willand N.; Markuszewski M.; Odell L. R.; Poelarends G. J.; De Spiegeleer B. Sustainability in Drug Discovery. Med. Drug Discovery 2021, 12 (June), 100107 10.1016/j.medidd.2021.100107.

[ref7] Castiello C.; Junghanns P.; Mergel A.; Jacob C.; Ducho C.; Valente S.; Rotili D.; Fioravanti R.; Zwergel C.; Mai A. GreenMedChem: The Challenge in the next Decade toward Eco-Friendly Compounds and Processes in Drug Design. Green Chem. 2023, 25 (6), 2109–2169. 10.1039/D2GC03772F.

[ref8] Martinengo B.; Diamanti E.; Uliassi E.; Bolognesi M. L. Harnessing the 12 Green Chemistry Principles for Sustainable Antiparasitic Drugs: Toward the One Health Approach. ACS Infect. Dis. 2024, 10 (6), 1856–1870. 10.1021/acsinfecdis.4c00172. 38724015 PMC11184551

[ref9] Bryan M. C.; Dillon B.; Hamann L. G.; Hughes G. J.; Kopach M. E.; Peterson E. A.; Pourashraf M.; Raheem I.; Richardson P.; Richter D.; Sneddon H. F. Sustainable Practices in Medicinal Chemistry: Current State and Future Directions. J. Med. Chem. 2013, 56 (15), 6007–6021. 10.1021/jm400250p. 23586692

[ref10] Aliagas I.; Berger R.; Goldberg K.; Nishimura R. T.; Reilly J.; Richardson P.; Richter D.; Sherer E. C.; Sparling B. A.; Bryan M. C. Sustainable Practices in Medicinal Chemistry Part 2: Green by Design. J. Med. Chem. 2017, 60 (14), 5955–5968. 10.1021/acs.jmedchem.6b01837. 28375009

[ref11] da S.M. Forezi L.; Ferreira P. G.; de Carvalho A. S.; de C da Silva F.; F V. Medicinal Chemistry for Sustainable Development. Curr. Top Med. Chem. 2023, 23 (11), 957–969. 10.2174/1568026623666230517114621. 37198981

[ref12] Extreme Poverty Can. Be Eradicated. Nature 2023, 618, 886–886. 10.1038/d41586-023-02098-3 37380695

[ref13] Bhutta Z. A.; Sommerfeld J.; Lassi Z. S.; Salam R. A.; Das J. K. Global Burden, Distribution, and Interventions for Infectious Diseases of Poverty. Infect. Dis. Poverty 2014, 3 (1), 1–7. 10.1186/2049-9957-3-21. 25110585 PMC4126350

[ref14] World Health Organization. Ending the Neglect to Attain the Sustainable Development Goals: A Road Map for Neglected Tropical Diseases 2021–2030.; 2020.

[ref15] Bolognesi M. L. Sustainable Anti-Trypanosomatid Drugs: An Aspirational Goal for Medicinal Chemistry, 1st ed.; Elsevier Inc., 2019; Vol. 52. 10.1016/bs.armc.2019.05.003.

[ref16] Yang G. J.; Zhou X. N. A New Formulation of Praziquantel to Achieve Schistosomiasis Elimination. Lancet Infect. Dis. 2023, 23 (7), 774–776. 10.1016/S1473-3099(23)00059-2. 36893783

[ref17] N’Goran E. K.; Odiere M. R.; Assandé Aka R.; Ouattara M.; Aka N. A. D.; Ogutu B.; Rawago F.; Bagchus W. M.; Bödding M.; Kourany-Lefoll E.; Tappert A.; Yin X.; Bezuidenhout D.; Badenhorst H.; Huber E.; Dälken B.; Haj-Ali Saflo O. Efficacy, Safety, and Palatability of Arpraziquantel (L-Praziquantel) Orodispersible Tablets in Children Aged 3 Months to 6 Years Infected with Schistosoma in Côte d’Ivoire and Kenya: An Open-Label, Partly Randomised, Phase 3 Trial. Lancet Infect. Dis. 2023, 23 (7), 867–876. 10.1016/S1473-3099(23)00048-8. 36893784

[ref18] Ding D.; Zhao Y.; Meng Q.; Xie D.; Nare B.; Chen D.; Bacchi C. J.; Yarlett N.; Zhang Y. K.; Hernandez V.; Xia Y.; Freund Y.; Abdulla M.; Ang K. H.; Ratnam J.; McKerrow J. H.; Jacobs R. T.; Zhou H.; Plattner J. J. Discovery of Novel Benzoxaborole-Based Potent Antitrypanosomal Agents. ACS Med. Chem. Lett. 2010, 1 (4), 165–169. 10.1021/ml100013s. 24900190 PMC4007846

[ref19] Betu Kumeso V. K.; Kalonji W. M.; Rembry S.; Valverde Mordt O.; Ngolo Tete D.; Prêtre A.; Delhomme S.; Ilunga Wa Kyhi M.; Camara M.; Catusse J.; Schneitter S.; Nusbaumer M.; Mwamba Miaka E.; Mahenzi Mbembo H.; Makaya Mayawula J.; Layba Camara M.; Akwaso Massa F.; Kaninda Badibabi L.; Kasongo Bonama A.; Kavunga Lukula P.; Mutanda Kalonji S.; Mariero Philemon P.; Mokilifi Nganyonyi R.; Embana Mankiara H.; Asuka Akongo Nguba A.; Kobo Muanza V.; Mulenge Nasandhel E.; Fifi Nzeza Bambuwu A.; Scherrer B.; Strub-Wourgaft N.; Tarral A. Efficacy and Safety of Acoziborole in Patients with Human African Trypanosomiasis Caused by Trypanosoma Brucei Gambiense: A Multicentre, Open-Label, Single-Arm, Phase 2/3 Trial. Lancet Infect. Dis. 2023, 23 (4), 463–470. 10.1016/S1473-3099(22)00660-0. 36460027 PMC10033454

[ref20] Clark M. J.; Miduturu C.; Schmidt A. G.; Zhu X.; Pitts J. D.; Wang J.; Potisopon S.; Zhang J.; Wojciechowski A.; Hann Chu J. J.; Gray N. S.; Yang P. L. GNF-2 Inhibits Dengue Virus by Targeting Abl Kinases and the Viral e Protein. Cell Chem. Biol. 2016, 23 (4), 443–452. 10.1016/j.chembiol.2016.03.010. 27105280 PMC4865888

[ref21] Li Z.; Liu H. Y.; He Z.; Chakravarty A.; Golden R. P.; Jiang Z.; You I.; Yue H.; Donovan K. A.; Du G.; Che J.; Tse J.; Che I.; Lu W.; Fischer E. S.; Zhang T.; Gray N. S.; Yang P. L. Discovery of Potent Degraders of the Dengue Virus Envelope Protein. Adv. Sci. 2024, 11, 2405829 10.1002/advs.202405829. PMC11516100 39145423

[ref22] Liu H. Y.; Li Z.; Reindl T.; He Z.; Qiu X.; Golden R. P.; Donovan K. A.; Bailey A.; Fischer E. S.; Zhang T.; Gray N. S.; Yang P. L. Broad-Spectrum Activity against Mosquito-Borne Flaviviruses Achieved by a Targeted Protein Degradation Mechanism. Nat. Commun. 2024, 15 (1), 1–14. 10.1038/s41467-024-49161-9. 38898037 PMC11187112

[ref23] Espinoza-Chávez R. M.; Salerno A.; Liuzzi A.; Ilari A.; Milelli A.; Uliassi E.; Bolognesi M. L. Targeted Protein Degradation for Infectious Diseases: From Basic Biology to Drug Discovery. ACS Bio Med. Chem. Au 2023, 3 (1), 32–45. 10.1021/acsbiomedchemau.2c00063. PMC10125329 37101607

[ref24] Chabner B. A.; Roberts T. G. Jr Chemotherapy and the War on Cancer Roberts et Al, 2005. Nat. Rev. Cancer 2005, 5, 65–72. 10.1038/nrc1529. 15630416

[ref25] Cohen P.; Cross D.; Jänne P. A. Kinase Drug Discovery 20 Years after Imatinib: Progress and Future Directions. Nat. Rev. Drug Discovery 2021, 20 (7), 551–569. 10.1038/s41573-021-00195-4. 34002056 PMC8127496

[ref26] Bedard P. L.; Hyman D. M.; Davids M. S.; Siu L. L. Small Molecules, Big Impact: 20 Years of Targeted Therapy in Oncology. Lancet 2020, 395 (10229), 1078–1088. 10.1016/S0140-6736(20)30164-1. 32222192

[ref27] Blanco M. J.; Gardinier K. M.; Namchuk M. N. Advancing New Chemical Modalities into Clinical Studies. ACS Med. Chem. Lett. 2022, 13 (11), 1691–1698. 10.1021/acsmedchemlett.2c00375. 36385931 PMC9661701

[ref28] Roskoski R. Classification of Small Molecule Protein Kinase Inhibitors Based upon the Structures of Their Drug-Enzyme Complexes. Pharmacol. Res. 2016, 103, 26–48. 10.1016/j.phrs.2015.10.021. 26529477

[ref29] Ayala-Aguilera C. C.; Valero T.; Lorente-Macías Á.; Baillache D. J.; Croke S.; Unciti-Broceta A. Small Molecule Kinase Inhibitor Drugs (1995–2021): Medical Indication, Pharmacology, and Synthesis. J. Med. Chem. 2022, 65 (2), 1047–1131. 10.1021/acs.jmedchem.1c00963. 34624192

[ref30] Lu X.; Smaill J. B.; Ding K. Medicinal Chemistry Strategies for the Development of Kinase Inhibitors Targeting Point Mutations. J. Med. Chem. 2020, 63 (19), 10726–10741. 10.1021/acs.jmedchem.0c00507. 32432477

[ref31] Han B.; Salituro F. G.; Blanco M. J. Impact of Allosteric Modulation in Drug Discovery: Innovation in Emerging Chemical Modalities. ACS Med. Chem. Lett. 2020, 11 (10), 1810–1819. 10.1021/acsmedchemlett.9b00655. 33062158 PMC7549105

[ref32] Hillebrand L.; Liang X. J.; Serafim R. A. M.; Gehringer M. Emerging and Re-Emerging Warheads for Targeted Covalent Inhibitors: An Update. J. Med. Chem. 2024, 67 (10), 7668–7758. 10.1021/acs.jmedchem.3c01825. 38711345

[ref33] Amrhein J. A.; Knapp S.; Hanke T. Synthetic Opportunities and Challenges for Macrocyclic Kinase Inhibitors. J. Med. Chem. 2021, 64 (12), 7991–8009. 10.1021/acs.jmedchem.1c00217. 34076436

[ref34] Sakamoto K. M.; Kim K. B.; Verma R.; Ransick A.; Stein B.; Crews C. M.; Deshaies R. J. Development of Protacs to Target Cancer-Promoting Proteins for Ubiquitination and Degradation. Mol. Cell. Proteomics 2003, 2 (12), 1350–1358. 10.1074/mcp.T300009-MCP200. 14525958

[ref35] Rodriguez-Gonzalez A.; Cyrus K.; Salcius M.; Kim K.; Crews C. M.; Deshaies R. J.; Sakamoto K. M. Targeting Steroid Hormone Receptors for Ubiquitination and Degradation in Breast and Prostate Cancer. Oncogene 2008, 27 (57), 7201–7211. 10.1038/onc.2008.320. 18794799 PMC5573236

[ref36] Desantis J.; Mammoli A.; Eleuteri M.; Coletti A.; Croci F.; Macchiarulo A.; Goracci L. PROTACs Bearing Piperazine-Containing Linkers: What Effect on Their Protonation State?. RSC Adv. 2022, 12 (34), 21968–21977. 10.1039/D2RA03761K. 36043064 PMC9361468

[ref37] Zheng Q.; Peacock D. M.; Shokat K. M. Drugging the Next Undruggable KRAS Allele-Gly12Asp. J. Med. Chem. 2022, 65 (4), 3119–3122. 10.1021/acs.jmedchem.2c00099. 35167298

[ref38] Arvinas. Arvinas Announces First-in-Human Dosing of ARV-102, an Investigational PROTAC® Protein Degrader for Neurodegenerative Disease. https://ir.arvinas.com/news-releases/news-release-details/arvinas-announces-first-human-dosing-arv-102-investigational (accessed 2025-02-13).

[ref39] Estrada A. A.; Sweeney Z. K. Chemical Biology of Leucine-Rich Repeat Kinase 2 (LRRK2) Inhibitors. J. Med. Chem. 2015, 58 (17), 6733–6746. 10.1021/acs.jmedchem.5b00261. 25915084

[ref40] Silva M. C.; Ferguson F. M.; Cai Q.; Donovan K. A.; Nandi G.; Patnaik D.; Zhang T.; Huang H. T.; Lucente D. E.; Dickerson B. C.; Mitchison T. J.; Fischer E. S.; Gray N. S.; Haggarty S. J. Targeted Degradation of Aberrant Tau in Frontotemporal Dementia Patient-Derived Neuronal Cell Models. Elife 2019, 8, 1–31. 10.7554/eLife.45457. PMC6450673 30907729

[ref41] Uliassi E.; Bolognesi M. L.; Milelli A. Targeting Tau Protein with Proximity Inducing Modulators: A New Frontier to Combat Tauopathies 2025, 10.1021/acsptsci.4c00733. PMC11915046 40109749

[ref42] Sut S.; Baldan V.; Faggian M.; Peron G.; Dall’Acqua S. Nutraceuticals, A New Challenge for Medicinal Chemistry. Curr. Med. Chem. 2016, 23 (28), 3198–3223. 10.2174/0929867323666160615104837. 27319583

[ref43] Cunha R. A. How Does Adenosine Control Neuronal Dysfunction and Neurodegeneration?. J. Neurochem. 2016, 139 (6), 1019–1055. 10.1111/jnc.13724. 27365148

[ref44] Arendash G. W.; Mori T.; Cao C.; Mamcarz M.; Runfeldt M.; Dickson A.; Rezai-Zadeh K.; Tan J.; Citron B. A.; Lin X.; Echeverria V.; Potter H. Caffeine Reverses Cognitive Impairment and Decreases Brain Amyloid-β Levels in Aged Alzheimer’s Disease Mice. JAD 2009, 17 (3), 661–680. 10.3233/JAD-2009-1087. 19581722

[ref45] Nelson K. M.; Dahlin J. L.; Bisson J.; Graham J.; Pauli G. F.; Walters M. A. The Essential Medicinal Chemistry of Curcumin. J. Med. Chem. 2017, 60 (5), 1620–1637. 10.1021/acs.jmedchem.6b00975. 28074653 PMC5346970

[ref46] Baell J. B.; Holloway G. A. New Substructure Filters for Removal of Pan Assay Interference Compounds (PAINS) from Screening Libraries and for Their Exclusion in Bioassays. J. Med. Chem. 2010, 53 (7), 2719–2740. 10.1021/jm901137j. 20131845

[ref47] Atanasov A. G.; Zotchev S. B.; Dirsch V. M.; et al. Natural Products in Drug Discovery: Advances and Opportunities. Nat. Rev. Drug Discov 2021, 20, 200–216. 10.1038/s41573-020-00114-z. 33510482 PMC7841765

[ref48] Zuin V. G.; Eilks I.; Elschami M.; Kümmerer K. Education in Green Chemistry and in Sustainable Chemistry: Perspectives towards Sustainability. Green Chem. 2021, 23 (4), 1594–1608. 10.1039/D0GC03313H.

[ref49] Barth M.; Michelsen G. Learning for Change: An Educational Contribution to Sustainability Science. Sustain. Sci. 2013, 8 (1), 103–119. 10.1007/s11625-012-0181-5.

[ref50] Petillion R. J.; Freeman T. K.; Mcneil W. S. United Nations Sustainable Development Goals as a Thematic Framework for an Introductory Chemistry Curriculum. J. Chem. Educ. 2019, 96 (12), 2845–2851. 10.1021/acs.jchemed.9b00307.

[ref51] International Master in Sustainable Drug Discovery. https://sustainabledrugdiscovery.eu/ (accessed 2024-12-08).

[ref52] University College London. MSc Sustainable Chemistry. https://www.ucl.ac.uk/chemistry/study-here/postgraduate-taught/msc-sustainable-chemistry (accessed 2025-02-13).

[ref53] United Nations Children’s Fund (UNICEF), the United Nations Development Programme (UNDP), the W. B. and the W. H. O. (WHO). TDR, the Special Programme for Research and Training in Tropical Diseases. https://tdr.who.int/ (accessed 2024-10-25).

[ref54] UCL School of Pharmacy. Reality Software Wins Awards for Innovation. https://www.ucl.ac.uk/pharmacy/news/2024/nov/ucl-virtual-reality-software-wins-awards-innovation (accessed 2024-12-08).

[ref55] University of Dundee. Drug Discovery Unit. https://www.dundee.ac.uk/life-sciences/research/drug-discovery-unit (accessed 2024-10-25).

[ref56] Huryn D. M.; Bolognesi M. L.; Young W. B. Medicinal Chemistry: Where Are All the Women?. ACS Med. Chem. Lett. 2017, 8 (9), 900–902. 10.1021/acsmedchemlett.7b00321. 28947931 PMC5601382

[ref57] MacDonald S. J. F. Medicinal Chemistry in Big Pharma Has a Gender Diversity Problem. Future Med. Chem. 2022, 14 (19), 1345–1347. 10.4155/fmc-2022-0185. 36097877

[ref58] Bolognesi M. L.; Ganamet K. L.; Liu H.; Poulsen S. A.; Georg G. I.; Wang S. Women in Medicinal Chemistry: Ad Maiora!. J. Med. Chem. 2020, 63 (5), 1777–1778. 10.1021/acs.jmedchem.0c00228. 32078314

[ref59] Blanco M. J.; Huryn D. M. Women in Medicinal Chemistry Special Issue. ACS Med. Chem. Lett. 2020, 11 (3), 210–211. 10.1021/acsmedchemlett.0c00083. 32184943 PMC7073892

[ref60] Borsari C.; Matagne B.; Goncharenko K.; Moreira R.; Auberson Y. P. The Facets of Diversity: The EFMC Perspective**. ChemMedChem. 2023, 18 (1), e202200245 10.1002/cmdc.202200245. 36538747

[ref61] Aldrich J.; Allen S.; Araujo E.; Bronson J.; Bryant-Friedrich A.; Cyr S. K.; DiMauro E. F.; Dzierba C.; Garner A. L.; Georg G. I.; Goodwin N. C.; Haranahalli K.; Huang R.; Leftheris K.; May-Dracka T. L.; Olson M. E.; Blanco M. J. Enhancing the Visibility of Women in the ACS Division of Medicinal Chemistry (ACS MEDI). J. Med. Chem. 2023, 66 (6), 3651–3655. 10.1021/acs.jmedchem.3c00350. 36884261

[ref62] Blanco M. J.; Bronson J. J.; DiMauro E. F.; Dzierba C.; Eggen M. J.; Garner A. L.; Georg G.; Giarolla J.; Goodwin N. C.; Grenier-Davies M. C.; Haskell-Luevano C.; Holzgrabe U.; Huang R.; Lagiakos H. R.; Leftheris K.; Martin Y.; Matos M. J.; May-Dracka T. L.; Müller C. E.; Newman A. H.; Parmee E.; Petter J. C.; Tamayo N. A.; Wexler R. R.; Bolognesi M. L.; Ripka A.; Young W. Empowering Voices: Inspiring Women in Medicinal Chemistry. J. Med. Chem. 2024, 67 (6), 4251–4258. 10.1021/acs.jmedchem.4c00493. 38456628

[ref63] Young W. B. Women’s Healthcare: Call for Action. J. Med. Chem. 2024, 67 (11), 8473–8480. 10.1021/acs.jmedchem.4c01135. 38804614

[ref64] Orive G.; Lertxundi U.; Brodin T.; Manning P. Greening the Pharmacy. Science (80-.) 2022, 377, 259–260. 10.1126/science.abp9554. 35857602

[ref65] (accessed 2024-11-19)

[ref66] Alfonsi K.; Colberg J.; Dunn P. J.; Fevig T.; Jennings S.; Johnson T. A.; Kleine H. P.; Knight C.; Nagy M. A.; Perry D. A.; Stefaniak M. Green Chemistry Tools to Influence a Medicinal Chemistry and Research Chemistry Based Organisation. Green Chem. 2008, 10 (1), 31–36. 10.1039/B711717E.

[ref67] Guillemard L.; Kaplaneris N.; Ackermann L.; Johansson M. J. Late-Stage C–H Functionalization Offers New Opportunities in Drug Discovery. Nat. Rev. Chem. 2021, 5 (8), 522–545. 10.1038/s41570-021-00300-6. 37117588

[ref68] Sharma P.; Ponnusamy E.; et al. DOZNTM 2.0: A Quantitative Green Chemistry Evaluator for a Sustainable Future. J. Organomet. Chem. 2022, 970–971, 122367 10.1016/j.jorganchem.2022.122367.

[ref69] Sherer E. C.; Bagchi A.; Kosjek B.; Maloney K. M.; Peng Z.; Robaire S. A.; Sheridan R. P.; Metwally E.; Campeau L. C. Driving Aspirational Process Mass Intensity Using Simple Structure-Based Prediction. Org. Process Res. Dev. 2022, 26 (5), 1405–1410. 10.1021/acs.oprd.1c00477.

[ref70] Gomollón-Bel F. Ten Chemical Innovations That Will Change Our World: IUPAC Identifies Emerging Technologies in Chemistry with Potential to Make Our Planet More Sustainable. Chem. Int. 2019, 41 ( (2), ). 12 10.1515/ci-2019-0203.

[ref71] Lin H.; Dai C.; Jamison T. F.; Jensen K. F. A Rapid Total Synthesis of Ciprofloxacin Hydrochloride in Continuous Flow. Angew. Chemie - Int. Ed. 2017, 56 (30), 8870–8873. 10.1002/anie.201703812. 28561939

[ref72] Xu C.; Nasrollahzadeh M.; Selva M.; Issaabadi Z.; Luque R. Waste-to-Wealth: Biowaste Valorization into Valuable Bio(Nano)Materials. Chem. Soc. Rev. 2019, 48 (18), 4791–4822. 10.1039/C8CS00543E. 31460520

[ref73] Espro C.; Paone E.; Mauriello F.; Gotti R.; Uliassi E.; Bolognesi M. L.; Rodríguez-Padrón D.; Luque R. Sustainable Production of Pharmaceutical, Nutraceutical and Bioactive Compounds from Biomass and Waste. Chem. Soc. Rev. 2021, 50 (20), 11191–11207. 10.1039/D1CS00524C. 34553208

[ref74] Pollini J.; Bragoni V.; Gooßen L. J. Synthesis of a Tyrosinase Inhibitor by Consecutive Ethenolysis and Cross-Metathesis of Crude Cashew Nutshell Liquid. Beilstein J. Org. Chem. 2018, 14, 2737–2744. 10.3762/bjoc.14.252. 30498524 PMC6244364

[ref75] Shi Y.; Kamer P. C. J.; Cole-Hamilton D. J. Synthesis of Pharmaceutical Drugs from Cardanol Derived from Cashew Nut Shell Liquid. Green Chem. 2019, 21 (5), 1043–1053. 10.1039/C8GC03823F.

[ref76] Soares Romeiro L. A.; Da Costa Nunes J. L.; De Oliveira Miranda C.; Simoies Heyn Roth Cardoso G.; De Oliveira A. S.; Gandini A.; Kobrlova T.; Soukup O.; Rossi M.; Senger J.; Jung M.; Gervasoni S.; Vistoli G.; Petralla S.; Massenzio F.; Monti B.; Bolognesi M. L. Novel Sustainable-by-Design HDAC Inhibitors for the Treatment of Alzheimer’s Disease. ACS Med. Chem. Lett. 2019, 10 (4), 671–676. 10.1021/acsmedchemlett.9b00071. 30996816 PMC6466821

[ref77] Rossi M.; Martinengo B.; Diamanti E.; Salerno A.; Rizzardi N.; Fato R.; Bergamini C.; Souza de Oliveira A.; de Araújo Marques Ferreira T.; Andrade Holanda C.; Romeiro L. A. S.; Soeiro M. de N. C.; Nunes K.; Ferreira de Almeida Fiuza L.; Meuser Batista M.; Fraga C. A. M.; E. A. Alkhalaf H.; Elmahallawy E. K.; Ebiloma G. U.; De Koning H. P.; Vittorio S.; Vistoli G.; Blanquart C.; Bertrand P.; Bolognesi M. L. Benign-by-Design SAHA Analogues for Human and Animal Vector-Borne Parasitic Diseases. ACS Med. Chem. Lett. 2024, 15 (9), 1506–1515. 10.1021/acsmedchemlett.4c00242. 39291036 PMC11403742

[ref78] Zhang Y.; Li Y. Global Decadal Assessment of Life below Water and on Land. iScience 2023, 26, 106420 10.1016/j.isci.2023.106420. 37035006 PMC10074189

[ref79] De Spiegeleer B.; Deprez B.; Hak E.; Markuszewski M.; Wynendaele E. On the Pivotal Role of Drug Discovery in Sustainable EU Pharma Reform. Drug Discovery Today 2024, 29 (10), 104097 10.1016/j.drudis.2024.104097. 38992421

[ref80] Brodin T.; Bertram M. G.; Arnold K. E.; Boxall A. B. A.; Brooks B. W.; Cerveny D.; Jörg M.; Kidd K. A.; Lertxundi U.; Martin J. M.; May L. T.; McCallum E. S.; Michelangeli M.; Tyler C. R.; Wong B. B. M.; Kümmerer K.; Orive G. The Urgent Need for Designing Greener Drugs. Nat. Sustain. 2024, 7, 949–951. 10.1038/s41893-024-01374-y.

[ref81] Patel M.; Kumar R.; Kishor K.; Mlsna T.; Pittman C. U.; Mohan D. Pharmaceuticals of Emerging Concern in Aquatic Systems: Chemistry, Occurrence, Effects, and Removal Methods. Chem. Rev. 2019, 119 (6), 3510–3673. 10.1021/acs.chemrev.8b00299. 30830758

[ref82] Kümmerer K. Sustainable from the Very Beginning: Rational Design of Molecules by Life Cycle Engineering as an Important Approach for Green Pharmacy and Green Chemistry. Green Chem. 2007, 9 (8), 899–907. 10.1039/b618298b.

[ref83] Maertens B. A. Green Chemistry for Green Toxicology. RSC Green Chem. 2022, 68, 1–30. 10.1039/9781839164392-00001.

[ref84] Lima C. M.; Uliassi E.; Thoré E. S. J.; Bertram M. G.; Cardoso L.; Cordeiro A.; Costi M. P.; Koning H. P. De. Environmental Impacts of Drugs against Parasitic Vector-Borne Diseases and the Need to Integrate Sustainability into Their Development and Use. Open Res. Eur. 2024, 4 (4), 207 10.12688/openreseurope.18008.1. 39534878 PMC11555358

[ref85] Kar S.; Sanderson H.; Roy K.; Benfenati E.; Leszczynski J. Ecotoxicological Assessment of Pharmaceuticals and Personal Care Products Using Predictive Toxicology Approaches. Green Chem. 2020, 22 (5), 1458–1516. 10.1039/C9GC03265G.

[ref86] Puhlmann N.; Vidaurre R.; Kümmerer K. Designing Greener Active Pharmaceutical Ingredients: Insights from Pharmaceutical Industry into Drug Discovery and Development. Eur. J. Pharm. Sci. 2024, 192, 106614 10.1016/j.ejps.2023.106614. 37858896

[ref87] Leder C.; Suk M.; Lorenz S.; Rastogi T.; Peifer C.; Kietzmann M.; Jonas D.; Buck M.; Pahl A.; Kümmerer K. Reducing Environmental Pollution by Antibiotics through Design for Environmental Degradation. ACS Sustain. Chem. Eng. 2021, 9 (28), 9358–9368. 10.1021/acssuschemeng.1c02243.

[ref88] Buchwald P.; Bodor N. Recent Advances in the Design and Development of Soft Drugs. Pharmazie 2014, 69 (6), 403–413. 10.1691/ph.2014.3911R. 24974571

[ref89] Bodor N.; Buchwald P. Soft Drug Design: General Principles and Recent Applications. Med. Res. Rev. 2000, 20 (1), 58–101. 10.1002/(SICI)1098-1128(200001)20:1<58::AID-MED3>3.0.CO;2-X. 10608921

[ref90] Chaganti T.; Tsai C. Y.; Juang Y. P.; Abdelalim M.; Cernak T. Medicinal Chemistry Gone Wild. J. Med. Chem. 2024, 67 (9), 6899–6905. 10.1021/acs.jmedchem.3c02334. 38662285

[ref91] WWF. Biodiversity Risk Filter. https://riskfilter.org/biodiversity/home (accessed 2024-11-21).

[ref92] Pathak V. M.; Verma V. K.; Rawat B. S.; Kaur B.; Babu N.; Sharma A.; Dewali S.; Yadav M.; Kumari R.; Singh S.; Mohapatra A.; Pandey V.; Rana N.; Cunill J. M. Current Status of Pesticide Effects on Environment, Human Health and It’s Eco-Friendly Management as Bioremediation: A Comprehensive Review. Front. Microbiol. 2022, 13, 1–29. 10.3389/fmicb.2022.962619. PMC9428564 36060785

[ref93] Johnson P. A. Agricultural and Pharmaceutical Chemicals. In Kirkwood R. C., Longley A. J. (eds) Clean Technology and the Environment. Springer, Dordrecht.; 1995. 10.1007/978-94-011-1312-0_7.

[ref94] Lamberth C. Latest Research Trends in Agrochemical Fungicides: Any Learnings for Pharmaceutical Antifungals?. ACS Med. Chem. Lett. 2022, 13 (6), 895–903. 10.1021/acsmedchemlett.2c00113. 35707143 PMC9190031

[ref95] EFMC. EFMC-ISMC 2024 - XXVIII EFMC International Symposium on Medicinal Chemistry. https://www.efmc-ismc.org/produits.php?langue=english&cle_menus=1238917591&cle_data=1360153628&output=4 (accessed 2024-12-09).

[ref96] B.Ethical. https://www.bethical.it/ (accessed 2024-12-09).

[ref97] Zafra Lerman E. Z. Malta conferences use science diplomacy as a bridge to peace in the Middle East. c&en Chemical & Engineering news. https://cen.acs.org/education/outreach/Malta-conferences-use-science-diplomacy/98/i10 (accessed 2024-12-09).

